# Supplementary material for: Electroluminescence and hyperphosphorescence from stable blue Ir(III) carbene complexes with suppressed efficiency roll-off
Source: Nat Commun. 2023 Oct 12;14:6419. doi: 10.1038/s41467-023-42090-z (PMC10570383; doi:10.1038/s41467-023-42090-z)
Supplement: Supplementary file 1 — Supplementary Information [file 41467_2023_42090_MOESM1_ESM.pdf]

Supplementary Information

Electroluminescence and hyperphosphorescence from stable blue Ir(III) carbene complexes with suppressed efficiency roll-off

Jie Yan[1,†], Dong-Ying Zhou[2,†], Liang-Sheng Liao[2]*, Martin Kuhn[3], Xiuwen Zhou[3]*, Shek-Man Yiu[4], and Yun Chi[1,4,5]*

[1]Department of Materials Science and Engineering, City University of Hong Kong, Hong Kong SAR 999077, China.

[2]Institute of Functional Nano and Soft Materials (FUNSOM), Jiangsu Key Laboratory for Carbon-Based Functional Materials & Devices, Soochow University, Suzhou 215123, China.

[3]School of Mathematics and Physics, The University of Queensland, Brisbane, Queensland 4072, Australia.

[4]Department of Chemistry, City University of Hong Kong, Hong Kong SAR 999077, China.

[5]Center of Super-Diamond and Advanced Films (COSDAF), City University of Hong Kong, Hong Kong SAR 999077, China.

[†]These authors contributed equally: Jie Yan, Dong-Ying Zhou

Email: lsliao@suda.edu.cn; x.zhou6@uq.edu.au; yunchi@cityu.edu.hk

Supplementary Note 1. General information and materials. Commercially available reagents were used without further purification. All solvents were dried and degassed before used, and all reactions were conducted under N_2 and monitored using precoated TLC plates (0.20 nm with fluorescent indicator F254). ^{1}H NMR spectra were recorded with Bruker 400 MHz "AVANCE III" Nuclear Magnetic Resonance System. Mass spectra were obtained on 4800 Plus MALDI T TOF/TOF Analyzer (ABI), where 2,5-dihydroxybenzoic acid was applied as the matrix. TGA measurements were performed on a TA Instrument TGAQ50, at a heating rate of 10 °C min^{-1} under a nitrogen atmosphere.

Supplementary Note 2. Photophysical measurements: All photophysical measurements in this study were performed at RT (298 K). UV-visible spectra were recorded on HITACHI

UH4150. The emission spectra of the solution state were measured with an EEM Fluorescence/Absorbance Spectrometer (Horiba-Duetta). Both wavelength-dependent excitation and emission responses of the fluorometer were calibrated. Absorption and emission spectra of the studied complexes were measured in dilute toluene at RT, and coumarin 102 was used as standard, where spectral grade solvents were employed. To specify the quantum yield in the fluid state, samples were degassed using at least three freeze-pump-thaw cycles. The solution quantum yields are calculated using coumarin 102 that has a known quantum yield, according to the following equation:

$$\Phi = \Phi_R \frac{I}{I_R} \frac{A_R}{A} \frac{\eta^2}{\eta_R^2} \quad (1)$$

Where Φ is the PL quantum yield, the subscript R refers to the reference compound of known quantum yield, I is the integrated fluorescence intensity, and η is the refractive index of solvent. A is the absorbance at the excitation wavelength with the measured absorbance between 0.05 – 0.1.

Supplementary Note 3. Electrochemistry: Cyclic voltammetry was conducted on a CHI660 Electrochemical Analyzer. Ag/Ag^+ (0.01 M $AgNO_3$) electrode was employed as the reference electrode. Oxidation and reduction potentials were measured using platinum working electrode with 0.1 M of NBu_4PF_6 as electrolyte in acetonitrile. The potentials were referenced externally to a ferrocenium/ferrocene (Fc^+/Fc) couple.

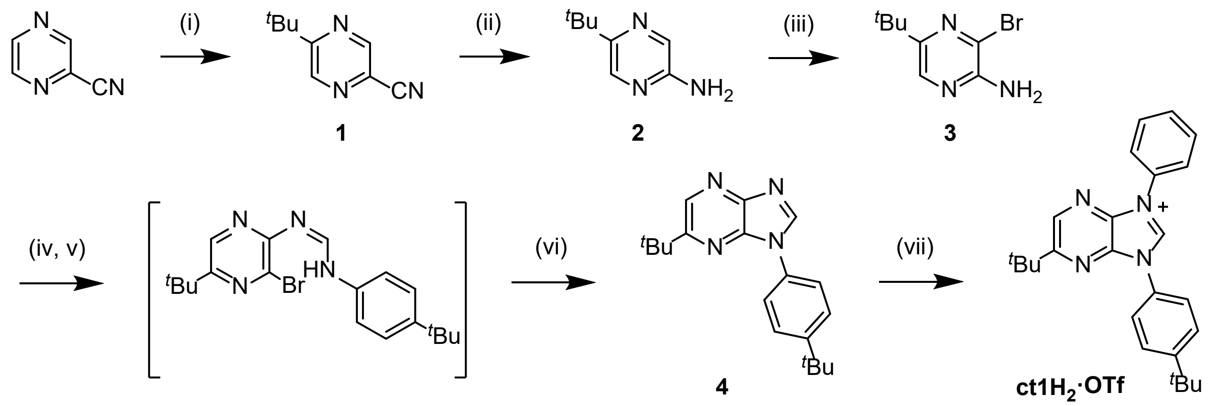

Supplementary Fig. 1. **Synthetic protocol to the imidazo[4,5-b]pyraz-3-ium pro-chelates**; experimental conditions: (i) pivalic acid, AgOTf, $(NH_4)_2S_2O_8$, 80 °C; (ii) NaOCl, NaOH, $^nBu_4NCl$, 80 °C; (iii) NBS, 0 °C; (iv) triethyl orthoformate, 140 °C; (v) 4-*t*-butyl aniline, 140 °C; (vi) DBU, CuI, 120 °C; (vii) $Cu(OAc)_2$, $(Ph_2I^+)(CF_3SO_3^-)$, 110 °C.

Supplementary Note 4. Synthesis of 5-(*t*-butyl)pyrazine-2-carbonitrile (1). To a 500 mL round-bottom flask was added pyrazinecarbonitrile (21.0 g, 0.2 mol) and deionized water (120 mL). An aqueous solution of silver triflate (0.17 M, 180 mL) and pivalic acid (22.4 g, 0.22 mol) was added in sequence. The resulting mixture was heated and stirred at 80 °C for 15 minutes. Then, ammonium peroxydisulfate (25.1 g, 0.11 mol) in water (70 mL) was added dropwise. The mixture was stirred for 12 hours and, then, cooled to RT. The mixture was neutralized with sodium carbonate and filtered, and precipitate was thoroughly extracted with ethyl acetate (EA). The organic phase was separated, washed with deionized water, and concentrated. The solid residue was further purified by column chromatography eluting with hexane to attain a white crystalline solid (24.5 g, 75%).

Selected spectral data: ^1H NMR (400 MHz, $CDCl_3$, 296 K): δ 8.8 (s, 1H), 8.78 (s, 1H), 1.43 (s, 9H).

Supplementary Note 5. Synthesis of 5-(*t*-butyl)pyrazin-2-amine (2). To a 500 mL two-necked flask was added 5-(*t*-butyl)pyrazine-2-carbonitrile (19.4 g, 0.12 mol), NaOH (24 g, 0.6 mmol) and tetra-n-butylammonium chloride in water (15 g, 50.7 mmol, 200 mL). The mixture was heated to 80 °C and, after then, NaOCl (14% aqueous solution, 200 mL) was slowly added, and the mixture was stirred for 12 hours. After cooled down to RT, the precipitate was taken into excessive ethyl acetate (EA). The organic phase was washed with deionized water, dried over anhydrous Na_2SO_4, concentrated and further purified by column chromatography eluting with a mixture of hexane/EA (3/1, v/v) to attain a red liquid (7.2 g, 40%).

Selected spectral data: ^{1}H NMR (400 MHz, $CDCl_3$, 296 K): δ 8.03 (s, 1H), 7.95 (s, 1H), 4.42 (br, 2H), 1.33 (s, 9H).

Supplementary Note 6. Synthesis of 3-bromo-5-(*t*-butyl)pyrazin-2-amine (3). To a 500 mL round flask was added 5-(*t*-butyl)pyrazin-2-amine (6.0 g, 39.6 mmol) and 250 mL of dichloromethane. After cooled to 0 °C, *N*-bromosuccinimide (NBS, 7.41 g, 41.7 mmol) was added slowly and the solution was stirred for 30 minutes. It was warmed up to RT and stirred for another 30 minutes and, the reaction was quenched by addition of water. The organic phase was separated and concentrated and, the residue was purified by column chromatography eluting with hexane and ethyl acetate (6/1, v/v) to attain a light-yellow solid (5.13 g, 56%).

Selected spectral data: ^{1}H NMR (400 MHz, $CDCl_3$): δ 7.95 (s, 1H), 4.96 (s, 2H), 1.34 (s, 9H).

Supplementary Note 7. Synthesis of 6-(*t*-butyl)-1-(4-(*t*-butyl)phenyl)-1H-imidazo[4,5-b]pyrazine (4). A mixture of 3-bromo-5-(*t*-butyl)pyrazin-2-amine (3.0 g, 13 mmol) and triethyl orthoformate (2.1 g, 14.3 mmol) were heated at 140 °C for 8 hours. After removal of volatiles under vacuum at RT, 4-*t*-butylaniline (1.9 g, 13 mmol) was added, and the mixture was heated at 140 °C for another 8 hours. Then, 1,8-diazabicyclo[5.4.0]undec-7-ene (DBU, 3.9 g, 26 mmol), CuI (0.5 g, 2.6 mmol) and DMSO (50 mL) were added and stirred at 120 °C overnight. After cooled to RT, the solvent was evaporated, the precipitate was taken into ethyl acetate and the resulting solution was washed with deionized water. The organic phase was separated, concentrated, and purified by column chromatography eluting with hexane and ethyl acetate (3/1, v/v) to attain a yellow crystalline solid (2.1 g, 54%).

Selected spectral data: ^{1}H NMR (400 MHz, $CDCl_3$, 296 K): δ 8.73 (s, 1H), 8.64 (s, 1H), 7.79 (d, *J* = 8.4 Hz, 2H), 7.63 (d, *J* = 8.4 Hz, 2H), 1.50 (s, 9H), 1.42 (s, 9H).

Supplementary Note 8. Synthesis of 6-(*t*-butyl)-3-phenyl-1-(4-(*t*-butyl)phenyl)-1H-imidazo[4,5-b]pyrazin-3-ium trifluoromethanesulfonate (ctu1H_2·OTf). To a 50 mL round-bottom flask was added 6-(*t*-butyl)-1-(4-(*t*-butyl)phenyl)-1H-imidazo[4,5-b]pyrazine (200 mg, 0.65 mmol), $Cu(OAc)_2 \cdot H_2O$ (6.5 mg, 0.03 mmol), diphenyliodonium triflate (413 mg, 0.97 mmol) and 10 mL DMF. After stirred at 110 °C for 6 hours, the solvent was concentrated by half under reduced pressure, followed by addition of deionized water to induce precipitation.

After then, the precipitate was collected and washed with deionized water and methanol in sequence, and dried under vacuum to attain an off-white powder (290 mg, 83%).

Spectral data of ctu1H_2·OTf: ^{1}H NMR (400 MHz, DMSO-d_6, 296 K): δ 11.06 (s, 1H), 9.22 (s, 1H), 8.02 (t, J = 5.6 Hz, 2H), 8.01 (d, J = 3.4 Hz, 2H), 7.86 – 7.71 (m, 5H), 1.50 (s, 9H), 1.40 (s, 9H).

Supplementary Note 9. Single X-Ray Structural Determination: The single crystals of f-ct1a, f-ct1b and f-ct1c suitable for X-ray diffraction study were obtained via the slow diffusion of methanol into the saturated CH_2Cl_2 solution at RT, respectively. In contrast, single crystals of f-ct1d were obtained from a mixed solution of methanol, CH_2Cl_2 and $CHCl_3$ at RT. Single crystal X-ray diffraction data were recorded on a Bruker D8 Venture Photon II diffractometer with microfocus X-ray sources using phi and omega scans mode (APEX3) at 233 K. All data have been deposited to the Cambridge Crystallographic Data Centre (CCDC) and can be obtained free of charge on application, 12 Union Road, Cambridge CB21EZ, UK (fax: (+44) 1223-336-033; e-mail: deposit@ccdc.cam.ac.uk).

Selected crystal data of f-ct1a: CCDC deposition number: 2170195. $C_{75}H_{81}N_{12}Ir$; M = 1342.71; trigonal; space group = R-3; a = 38.484(8) Å, b = 38.484(8) Å, c = 24.409(5) Å; V = 31307(15) Å^3; Z = 18; ρ_{Calcd} = 1.282 g·cm^{-3}; F(000) = 12456.0, crystal size = 0.21 × 0.12 × 0.04 mm^3; λ(Mo K_α) = 0.71073 Å; T = 193(2) K; μ = 4.098 mm^{-1}; 77098 reflections collected, 14251 independent reflections (R_{int} = 0.0480, R_{sigma} = 0.0393), max. and min. transmission = 0.754 and 0.440, data / restraints / parameters = 14251 / 180 / 850, GOF = 1.004, final $R_1[I > 2\sigma(I)]$ = 0.0276 and wR_2(all data) = 0.0739.

Selected crystal data of f-ct1b: CCDC deposition number: 2170111. $C_{75.5}H_{82}IrN_{12}Cl$; M = 1385.18; monoclinic; space group = $P2_1/c$; a = 13.9005(4) Å, b = 26.4544(7) Å, c = 19.8474(5) Å; β = 100.5410(10)°; V = 7175.3(3) Å^3; Z = 4; ρ_{Calcd} = 1.282 g·cm^{-3}; F(000) = 2852.0, crystal size = 0.36 × 0.14 × 0.03 mm^3; λ(Mo K_α) = 0.71073 Å; T = 193(2) K; μ = 1.947 mm^{-1}; 109353 reflections collected, 14684 independent reflections (R_{int} = 0.0622, R_{sigma} = 0.0358), max. and min. transmission = 0.745 and 0.572, data / restraints / parameters = 14684 / 527 / 922, GOF = 1.053, final $R_1[I > 2\sigma(I)]$ = 0.0381 and wR_2(all data) = 0.1368.

Selected crystal data of f-ct1c: CCDC deposition number: 2170110. $C_{77}H_{85}IrN_{12}Cl_4$; M = 1512.56; triclinic; space group = P-1; a = 13.1528(4) Å, b = 14.6232(5) Å, c = 19.0628(6) Å; α = 84.9520(10)°, β = 88.8440(10)°, γ = 89.2910(10)°; V = 3651.3(2) Å^3; Z = 2; ρ_{Calcd} = 1.376 g·cm^{-3}; F(000) = 1552.0, crystal size = 0.43 × 0.21 × 0.03 mm^3; λ(Mo K_α) = 0.71073 Å; T = 193(2) K; μ =

2.026 mm^{-1}; 56172 reflections collected, 14929 independent reflections (R_{int} = 0.0464, R_{sigma} = 0.0422), max. and min. transmission = 0.745 and 0.642, data / restraints / parameters = 14929 / 106 / 892, GOF = 1.030, final $R_1[I > 2\sigma(I)]$ = 0.0297 and wR_2(all data) = 0.0738.

Selected crystal data of f-ct1d: CCDC deposition number: 2198299. $C_{76}H_{82}IrN_{12}Cl_3$; M = 1462.08; cubic; space group = I-43d; *a* = *b* = *c* = 31.3192(6) Å; V = 30720.8(18) Å^3; Z = 16; ρ_{Calcd} = 1.264 g·cm^{-3}; F(000) = 12000.0, crystal size = 0.42 × 0.16 × 0.04 mm^3; λ(Cu K_α) = 1.54178 Å; T = 193(2) K; μ = 4.692 mm^{-1}; 125155 reflections collected, 5253 independent reflections (R_{int} = 0.0622, R_{sigma} = 0.0216), max. and min. transmission = 0.754 and 0.531, data / restraints / parameters = 5253/249/359, GOF = 1.048, final $R_1[I > 2\sigma(I)]$ = 0.0401 and wR_2(all data) = 0.1105.

Supplementary Note 10. Procedures for sodium acetate and *p*-toluenesulfonic acid induced isomerization.

A degassed 1,2,4-trichlorobenzene (10 mL) solution of f-ct1a (50 mg, 0.038 mmol), sodium acetate (62 mg, 0.76 mmol) and p-toluenesulfonic acid (1.3 mg, 0.0076 mmol) was refluxed for 36 hours. After removal of solvent, the residue was taken into CH_2Cl_2 solution. The organic phase was washed with deionized water, separated, and concentrated to dryness. The residue was separated by column chromatography eluting with n-hexane and ethyl acetate (7/1, v/v), followed by recrystallization to obtain a yellow solid f-ct1a (2 mg, 4%), a yellow solid f-ct1b (18 mg, 36%), a light-yellow solid f-ct1c (22 mg, 44%) and a light-yellow solid f-ct1d (4 mg, 8%).

A degassed 1,2,4-trichlorobenzene (10 mL) solution of f-ct1b (50 mg, 0.038 mmol), sodium acetate (62 mg, 0.76 mmol) and p-toluenesulfonic acid (1.3 mg, 0.0076 mmol) was refluxed for 36 hours. After removal of solvent, the residue was taken into CH_2Cl_2 solution. The organic phase was washed with deionized water, separated, and concentrated to dryness. This gave a mixture of four f-stereoisomers The residue was separated by column chromatography eluting with n-hexane and ethyl acetate (7/1, v/v), followed by recrystallization to obtain a yellow solid f-ct1a (2 mg, 4%), a yellow solid f-ct1b (20 mg, 40 %), a light-yellow solid f-ct1c (23 mg, 42%) and a light-yellow solid f-ct1d (2 mg, 4%).

A degassed 1,2,4-trichlorobenzene (10 mL) solution of f-ct1c (50 mg, 0.038 mmol), sodium acetate (62 mg, 0.76 mmol) and p-toluenesulfonic acid (1.3 mg, 0.0076 mmol) was refluxed for 36 hours. After removal of solvent, the residue was taken into CH_2Cl_2 solution. The organic phase was washed with deionized water, separated, dried over anhydrous Na_2SO_4

and concentrated to dryness. This gave a mixture of four f-stereoisomers. The residue was separated by column chromatography eluting with n-hexane and ethyl acetate (7/1, v/v), followed by recrystallization to obtain a yellow solid f-ct1a (1 mg, 2%), a yellow solid f-ct1b (19 mg, 38%), a light-yellow solid f-ct1c (24 mg, 48%) and light-yellow solid f-ct1d (1 mg, 2%).

A degassed 1,2,4-trichlorobenzene (10 mL) solution of f-ct1d (50 mg, 0.038 mmol), sodium acetate (62 mg, 0.76 mmol) and p-toluenesulfonic acid (66 mg, 1.3 mg, 0.0076 mmol) was refluxed for 48 hours. After removal of solvent, the residue was taken into CH_2Cl_2 solution. The organic phase was washed with deionized water, separated, dried over anhydrous Na_2SO_4 and concentrated to dryness. This gave a mixture of four f-stereoisomers. The residue was separated by column chromatography eluting with n-hexane and ethyl acetate (7/1, v/v), followed by recrystallization to obtain a yellow solid f-ct1a (4 mg, 8%), a yellow solid f-ct1b (10 mg, 20 %), a light-yellow solid f-ct1c (12 mg, 24%) and. a light-yellow solid f-ct1d (22 mg, 44%).

Supplementary Note 11. Additional computational details and results of theoretical investigations:

Relativistic TD-DFT calculations were further performed to obtain more detailed information on spin-mixed electron excitations (e.g., the oscillator strength and the radiative rate for $S_0 \rightarrow T_1$ excitations) with ADF (2019 version)[1] using the B3LYP functional and Slater type TZP[2,3] basis sets. The COnductor-like Screening MOdel (COSMO)[4] is used to model solvent effects.

In these calculations, spin-orbit coupling (SOC) was added perturbatively[5] to one-component TDDFT[6] utilizing the one-component zeroth order regular approximation (ZORA).[7,8] A total of 24 spin-mixed excitations were calculated. The radiative rate (k_r) of an excited state was then calculated according to[9-11]

$$k_r = 2\epsilon^2 f / c^3 \qquad (2)$$

where ϵ is the excitation energy, f is the corresponding oscillate strength for the electronic excitation from the ground state to excited state, c represents the speed of light in vacuum and the equation is in atomic units. The three lowest energy states found in the SOC TDDFT calculations for the complexes considered here are derived from the three substrates in a triplet state with a small singlet admixture due to SOC.[12,13] There will be small energy differences between the three substates because of their different admixtures with the singlet state, the so called zero-field splitting (ZFS). As the ZFS is small compared to k_BT at

room temperature, the radiative rate of the triplet state is then calculated as an average over the three substrates within the assumption of fast thermalization.

Supplementary Note 12. Device fabrication and measurement. Phosphorescent and hyper-OLEDs were fabricated on patterned ITO glass substrates with a sheet resistance of 15 Ω $square^{-1}$. All the organic materials are supplied by Lumtec. Co. Ltd. (Taiwan) and were deposited by thermal evaporation under a vacuum pressure of 4.0×10^{-4} Pa. The evaporation rate and thickness of the films were controlled using a calibrated quartz crystal microbalance during deposition. The evaporation rates for organic films were at the range of 0.1 ~ 2.0 Å s^{-1} while for the aluminium 2 ~ 5 Å s^{-1}. After the deposition process, the OLEDs were encapsulated with a glass lid and UV-curable resin. The active area of all the devices was 10 mm^2. The current density–voltage (J-V) characteristics and luminance (with electroluminescence spectra) characteristics of all fabricated devices were evaluated using a home-made system, comprising a source meter (Keithley 2400) and spectroradiometer (Photoresearch 650). The measurements were conducted in ambient air at room temperature (298 K). The transient electroluminescence analysis of the fabricated devices was performed at a driving voltage of 6 V pulse with a width of 5 ms.

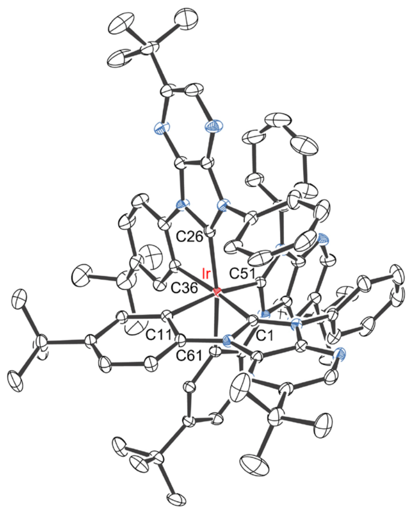

Supplementary Fig. 2. Structure of f-ct1a with thermal ellipsoids shown at 30% probability level. Selected bond lengths (Å): Ir1-C1 = 2.0317(18), Ir1-C26 = 2.029(2), Ir1-C51 = 2.0300(19), Ir1-C11 = 2.0766(19), Ir1-C36 = 2.0963(19), Ir1-C61 = 2.0983(19). Selected bond angles (°): C26-Ir1-C61 = 168.83(7), C1-Ir1-C36 = 172.52(7), C51-Ir1-C11 = 166.77(8). Hydrogen atoms were omitted for clarity.

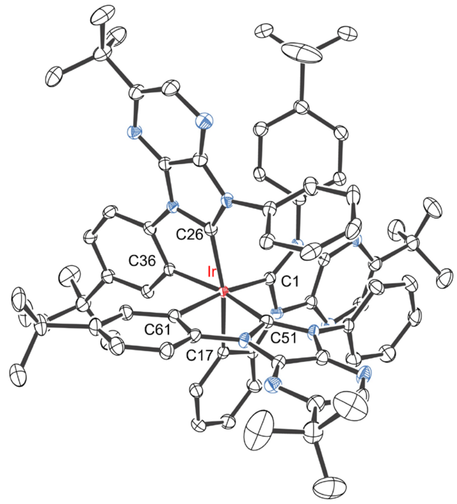

Supplementary Fig. 3. Structure of f-ct1b with thermal ellipsoids shown at 30% probability level. Selected bond lengths (Å): Ir1-C1 = 2.042(4), Ir1-C26 = 2.034(4), Ir1-C51 = 2.031(4), Ir1-C17 = 2.087(4), Ir1-C36 = 2.082(4), Ir1-C61 = 2.111(4). Selected bond angles (°): C26-Ir1-C17 = 167.77(17), C1-Ir1-C61 = 172.53(15), C51-Ir1-C36 = 166.75(16). Hydrogen atoms were omitted for clarity.

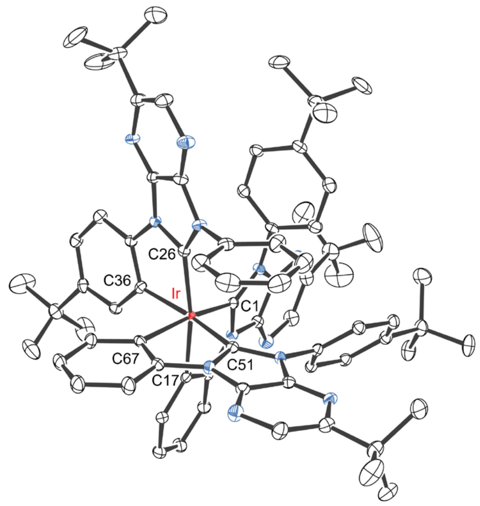

Supplementary Fig. 4. Structure of f-ct1c with thermal ellipsoids shown at 30% probability level. Selected bond lengths (Å): Ir1-C1 = 2.038(3), Ir1-C26 = 2.028(3), Ir1-C51 = 2.032(3), Ir1-C17 = 2.088(3), Ir1-C36 = 2.091(3), Ir1-C67 = 2.088(3). Selected bond angles (°): C26-Ir1-C17 = 168.05(11), C1-Ir1-C67 = 168.34(11), C51-Ir1-C36 = 170.36(10). Hydrogen atoms were omitted for clarity.

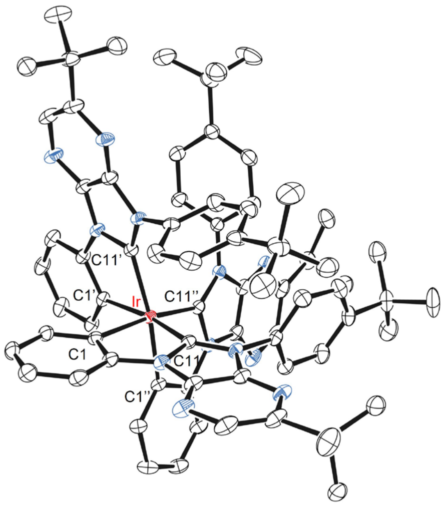

Supplementary Fig. 5. Structure of f-ct1d with thermal ellipsoids shown at 30% probability level. Selected bond lengths (Å): Ir1-C1 = 2.104(6), Ir1-C11 = 2.050(6). Selected bond angles (°): C1-Ir1-C11 = 77.9(3), C1-Ir1-C11'' = 170.3(3). Hydrogen atoms were omitted for clarity.

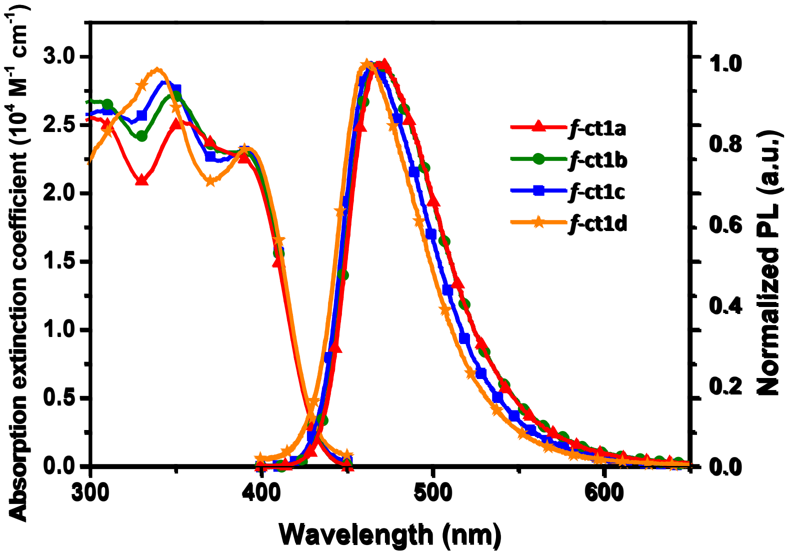

Supplementary Fig. 6. UV-Vis absorption and photoluminescence spectra of Ir(III) complexes f-ct1a – d in toluene at RT.

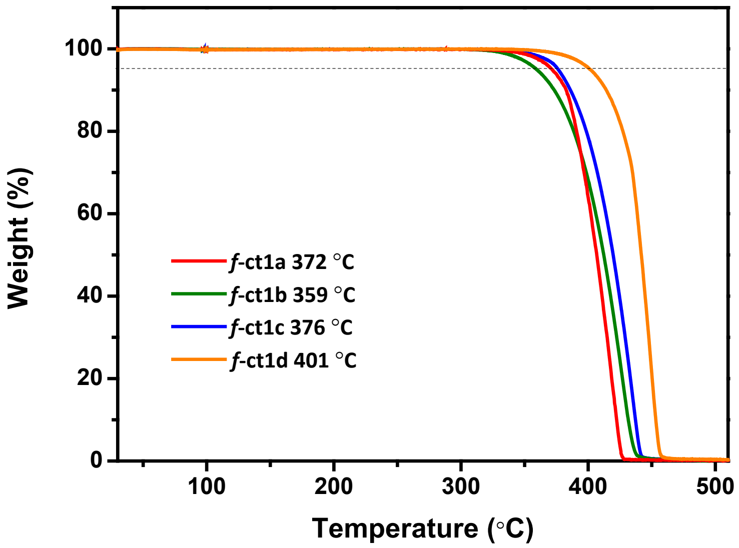

Supplementary Fig. 7. TGA data of studied Ir(III) complexes with decomposition temperature (T_d) at a weight loss of 5 wt.%.

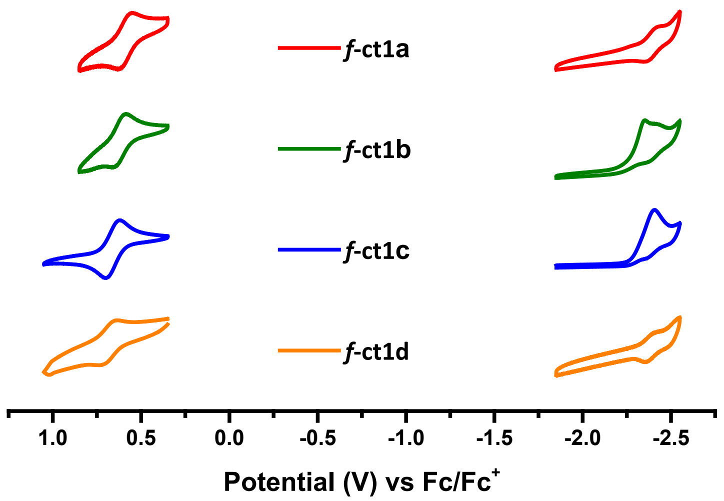

Supplementary Fig. 8. Cyclic voltammetric diagram of the studied Ir(III) complexes f-ct1a – d.

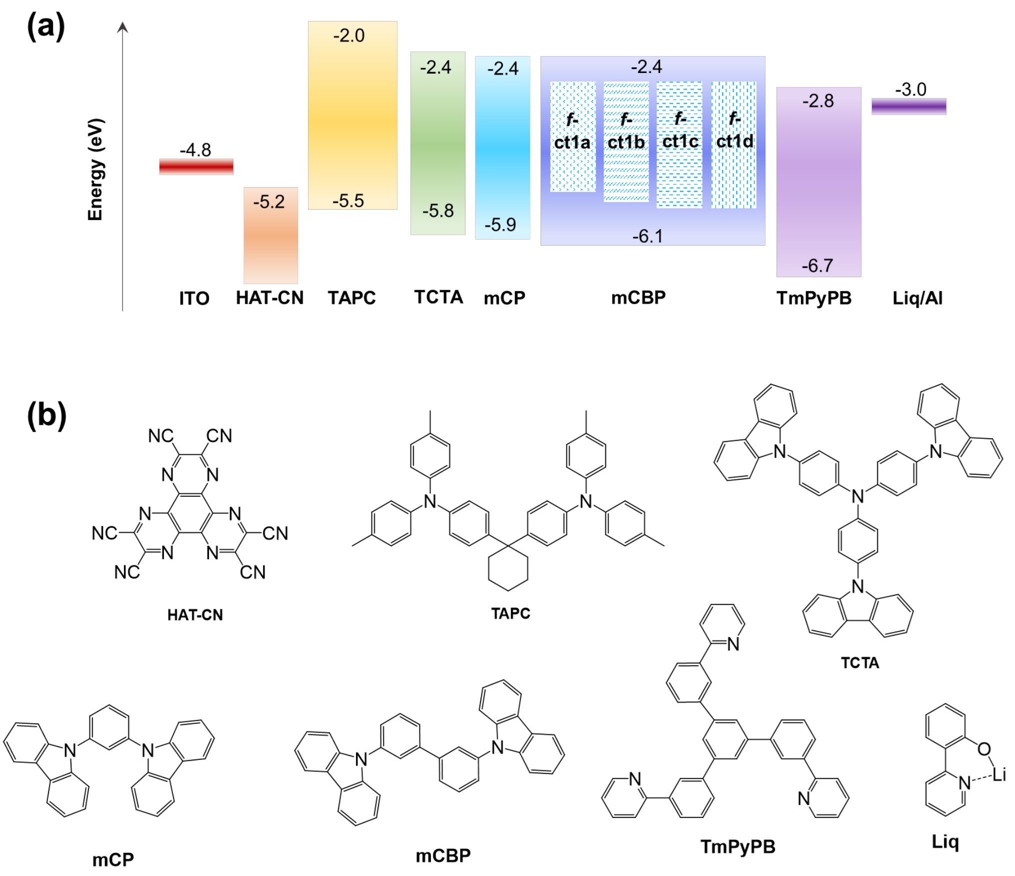

Supplementary Fig. 9. (a) Electron energy levels, (b) Molecular structures of the materials used in OLEDs.

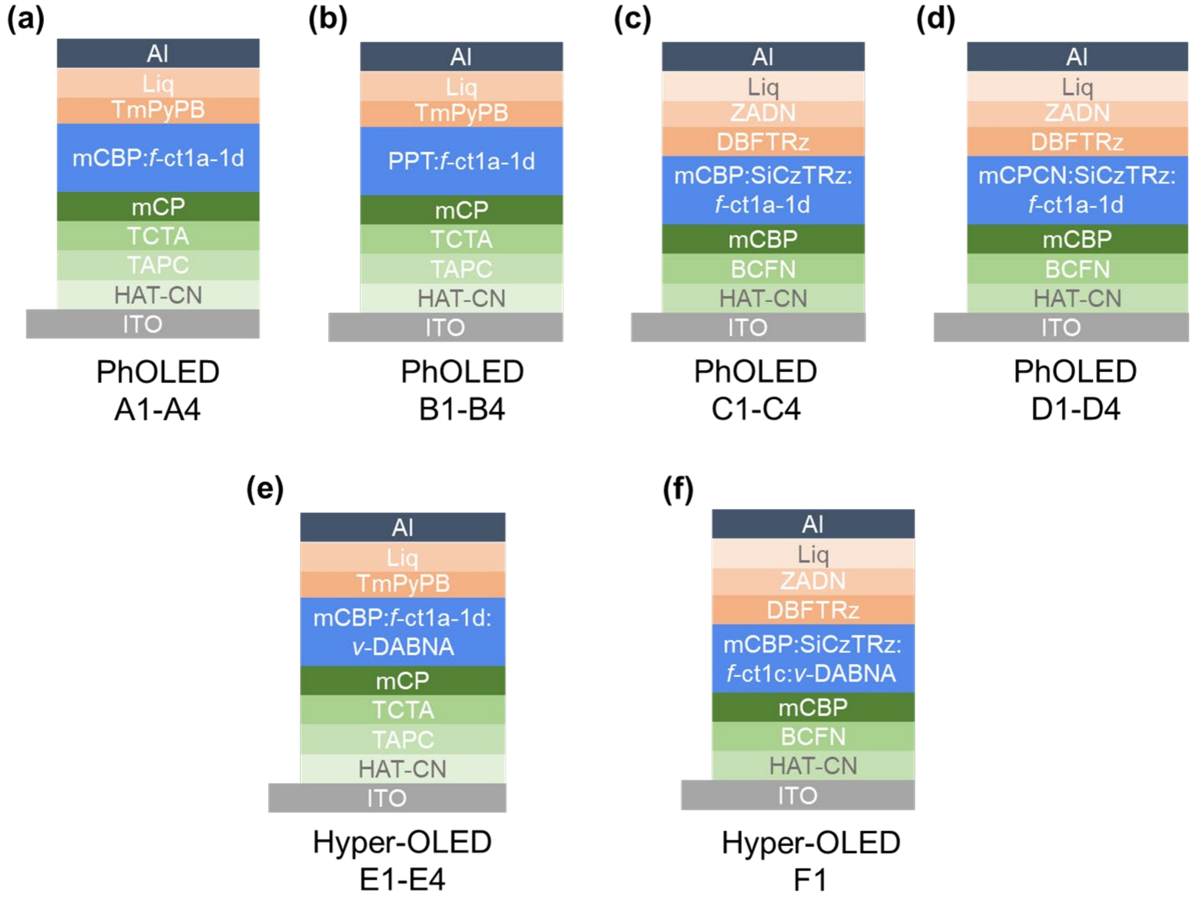

Supplementary Fig. 10. Schematic structures of OLEDs based on different architectures. (a) Schematic diagram of the OLED devices by using mCBP as host; (b) Schematic diagram of the OLED devices by using PPT as host; (c) Schematic diagram of the OLED devices by using mCBP and SiCzTRz as co-host; (d) Schematic diagram of the OLED devices by using mCPCN and SiCzTRz as co-host; (e) Schematic diagram of the Hyper-OLED devices by using mCBP as host; (f) Schematic diagram of the Hyper-OLED devices by using mCBP and SiCzTRz as co-host.

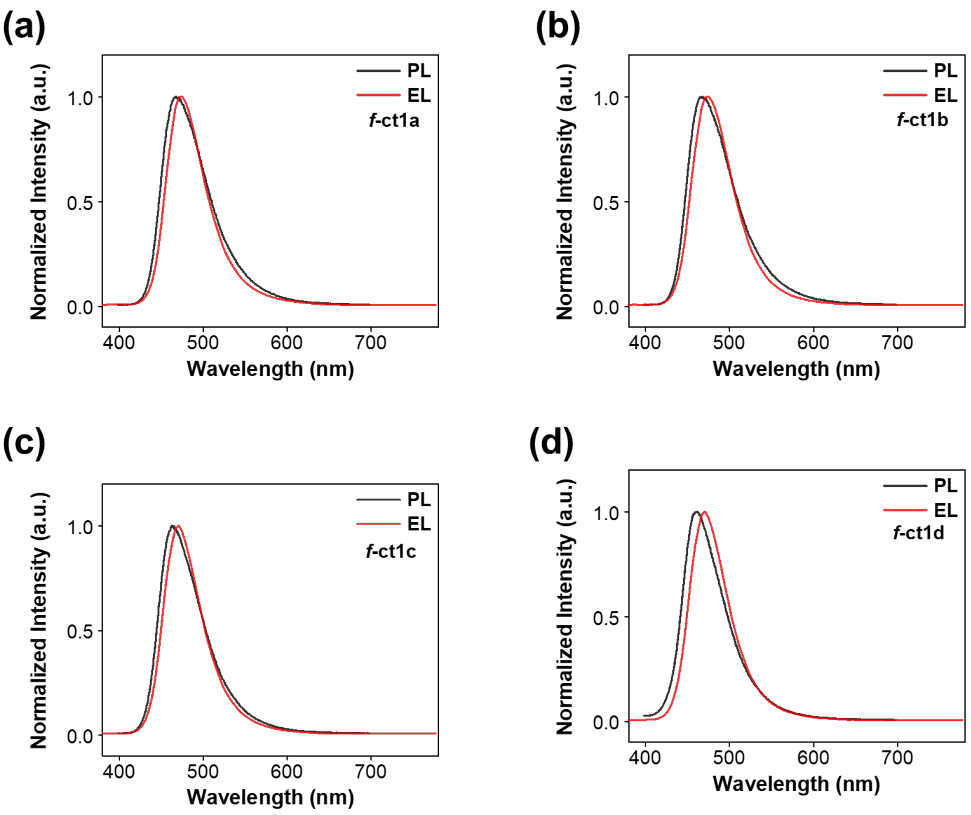

Supplementary Fig. 11. PL (in toluene solution) and EL spectra of (a) f-ct1a, (b) f-ct1b, (c) f-ct1c, and (d) f-ct1d.

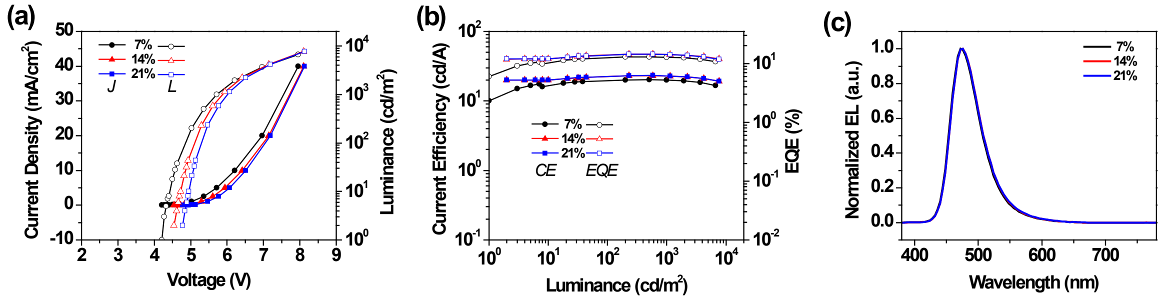

Supplementary Fig. 12. (a) Current density-voltage-luminance (J-V-L) characteristics; (b) Current efficiency-luminance-EQE characteristics; (c) Electroluminescence spectra of devices using f-ct1a as the emitter with different doping concentrations.

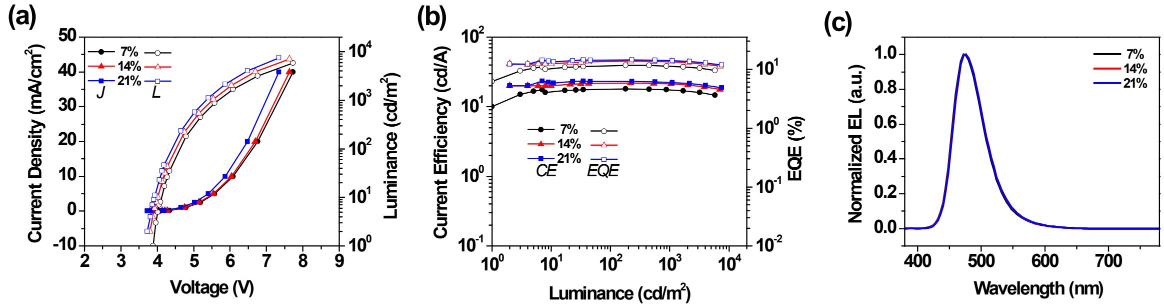

Supplementary Fig. 13. (a) Current density-voltage-luminance (J-V-L) characteristics; (b) Current efficiency-luminance-EQE characteristics; (c) Electroluminescence spectra of devices using f-ct1b as the emitter with different doping concentrations.

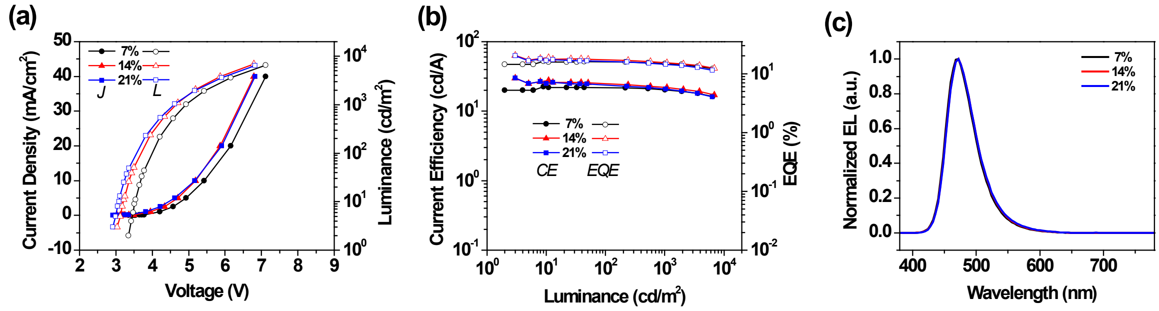

Supplementary Fig. 14. (a) Current density-voltage-luminance (J-V-L) characteristics; (b) Current efficiency-luminance-EQE characteristics; (c) Electroluminescence spectra of devices using f-ct1c as the emitter with different doping concentrations.

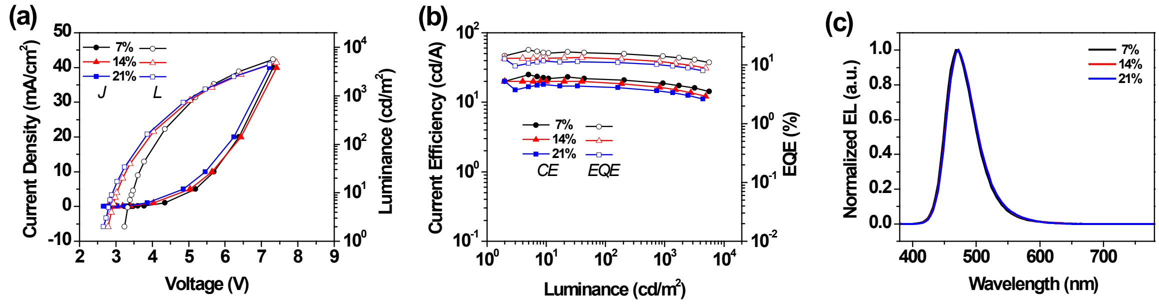

Supplementary Fig. 15. (a) Current density-voltage-luminance (J-V-L) characteristics; (b) Current efficiency-luminance-EQE characteristics; (c) Electroluminescence spectra of devices using f-ct1d as the emitter with different doping concentrations.

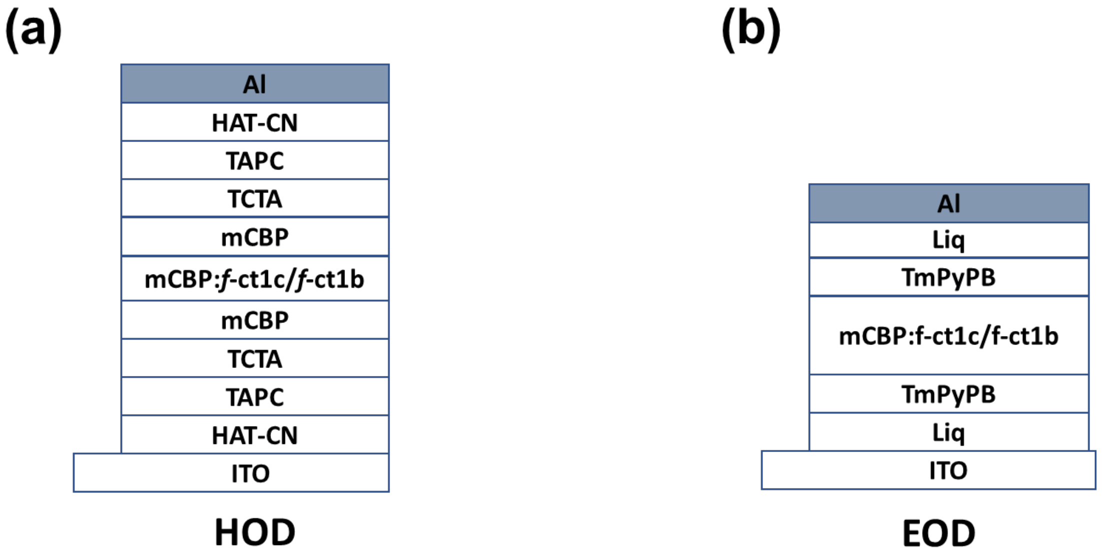

Supplementary Fig. 16. Schematic structure of (a) hole-only and (b) electron-only devices.

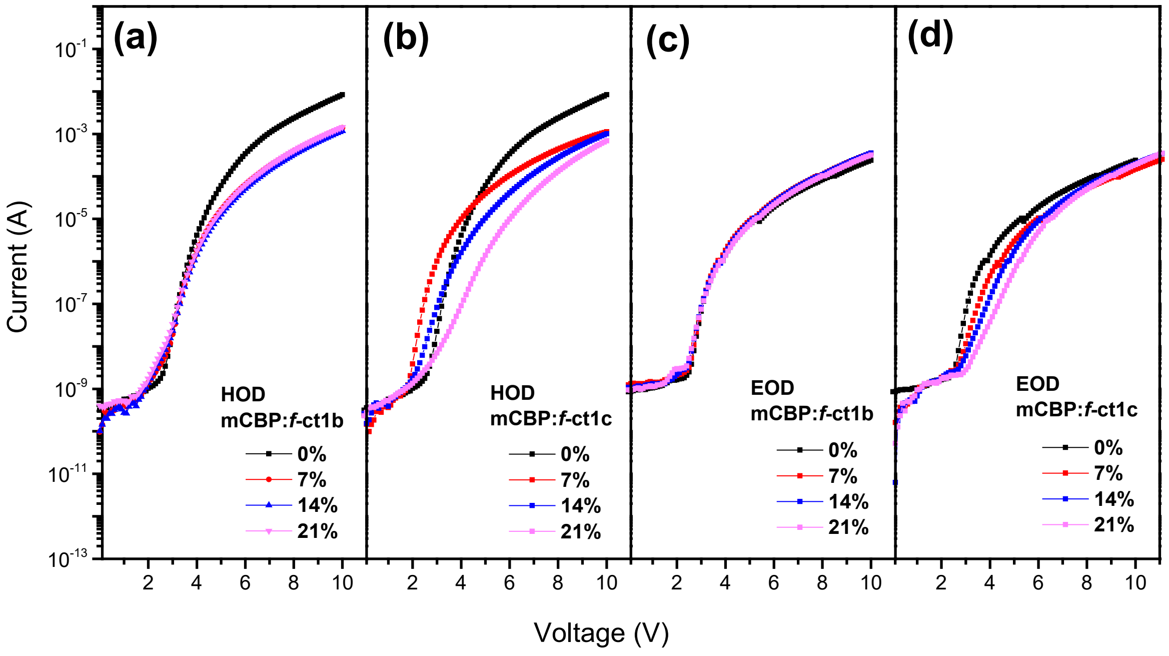

Supplementary Fig. 17. Current density versus voltage characteristics of hole-only devices based on different doping ratios of (a) f-ct1b and (b) f-ct1c, and (c) electron-only devices of (c) f-ct1b and (d) f-ct1c.

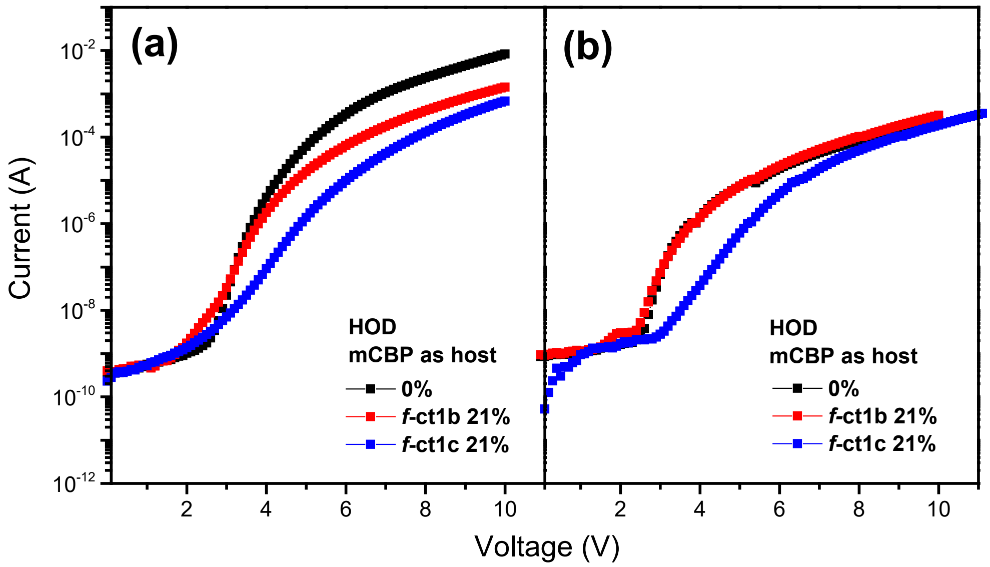

Supplementary Fig. 18. Current density versus voltage characteristics of (a) hole-only devices and (b) electron-only devices based on 21 wt% of f-ct1b and f-ct1c.

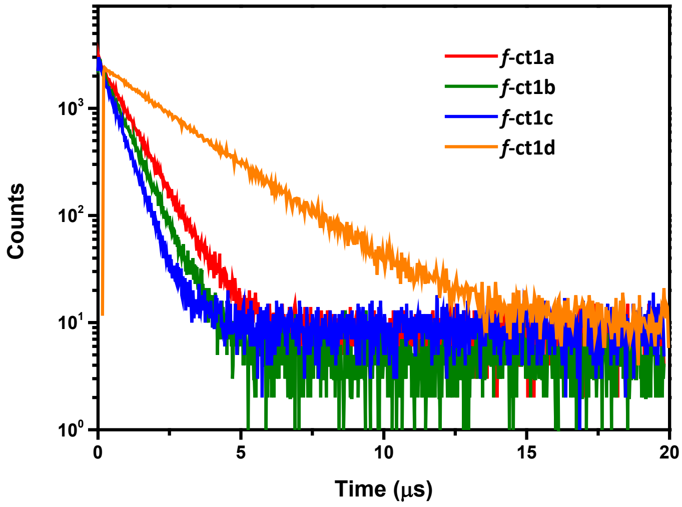

Supplementary Fig. 19. Transient PL decay curves of all studied Ir(III) complexes in degassed toluene at RT.

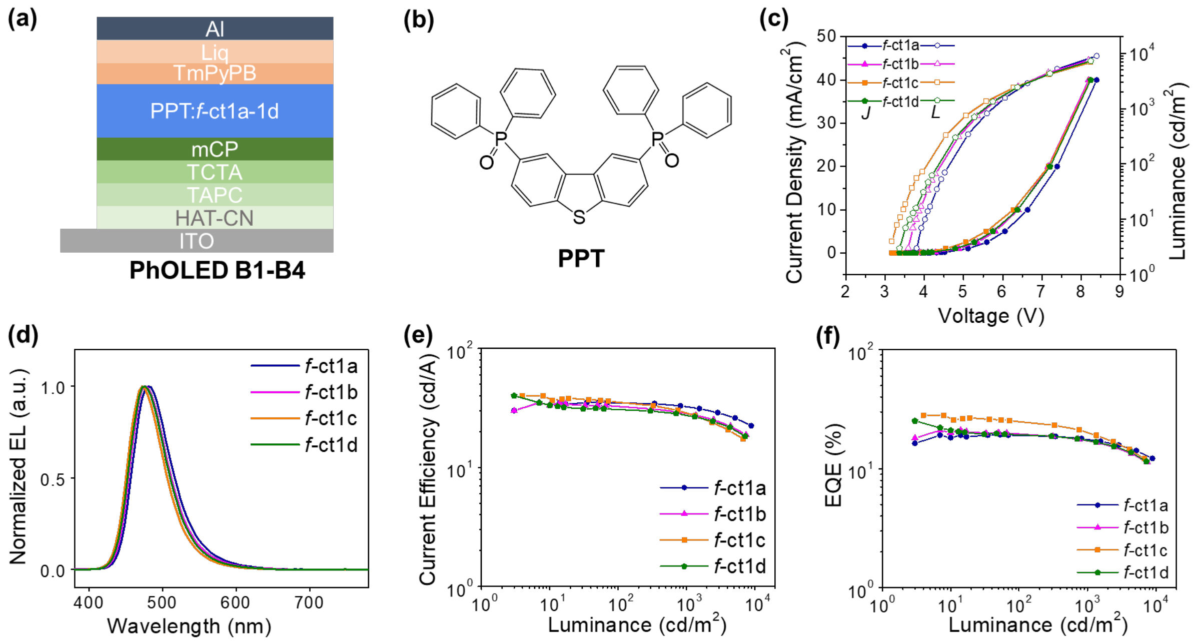

Supplementary Fig. 20. (a) Schematic diagram of the OLED devices; (b) Molecular structure of PPT; (c) Current density-voltage-luminance (J-V-L) characteristics; (d) EL spectra, (e) CE-luminance characteristics; (f) EQE-luminance characteristics. All doping conc. of Ir(III) complexes is maintained at 21 wt%.

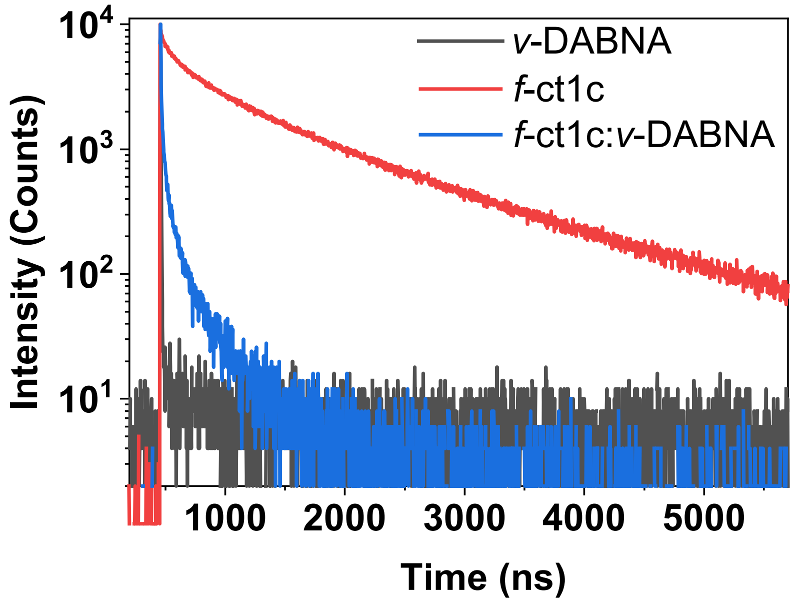

Supplementary Fig. 21. Transient PL traces of co-doped mCBP films with different dopants (1 wt% *v*-DABNA, 21 wt% f-ct1c, and 21 wt% f-ct1c:1 wt% *v*-DABNA) under the excitation of 375 nm pulsed laser.

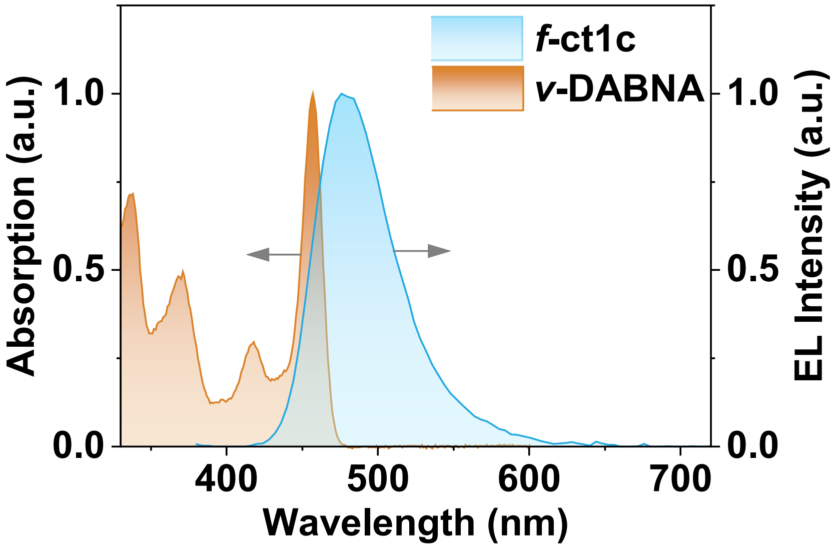

Supplementary Fig. 22. UV-Vis absorption spectrum of v-DABNA in toluene and EL spectrum of f-ct1c.

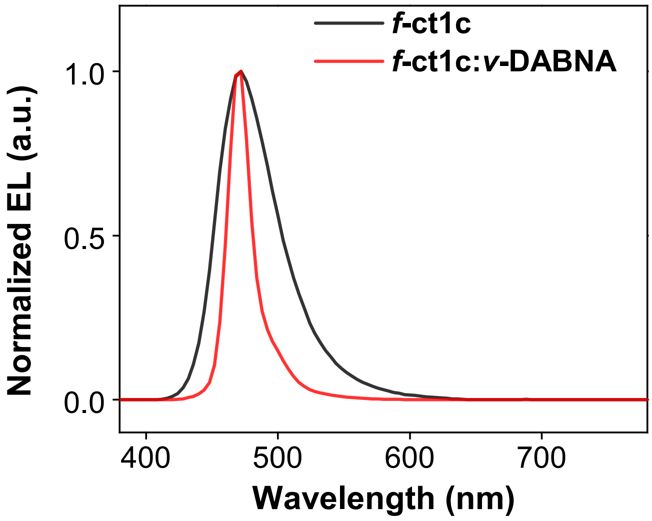

Supplementary Fig. 23. EL spectra of v-DABNA and a mixture of v-DABNA and f-ct1c.

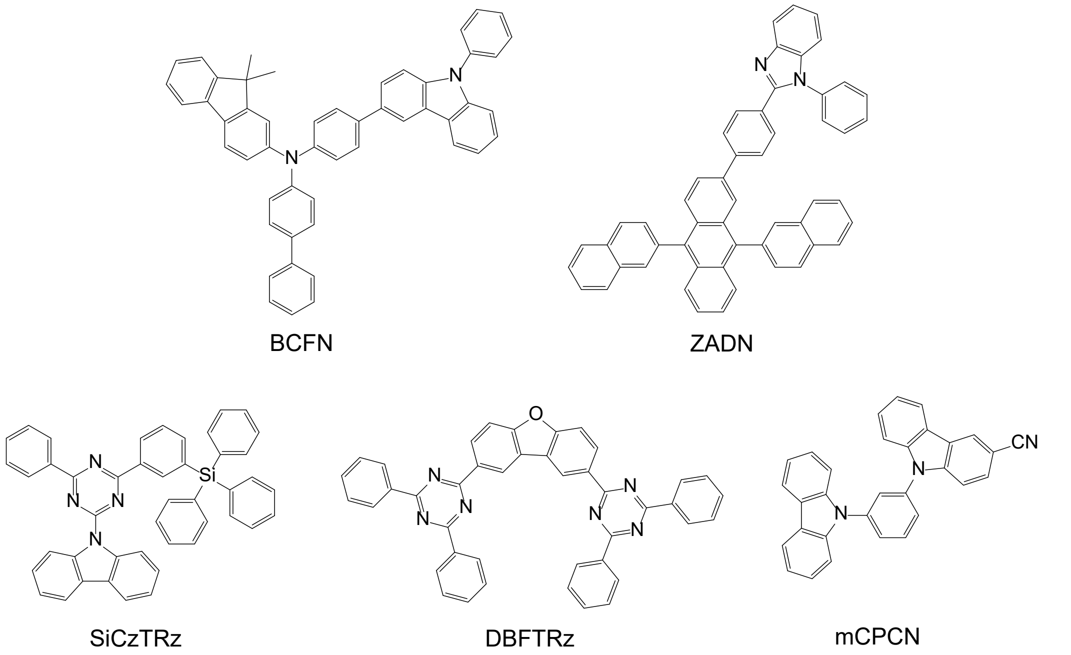

Supplementary Fig. 24. Molecular structures of the materials used in OLEDs for device lifetime measurement.

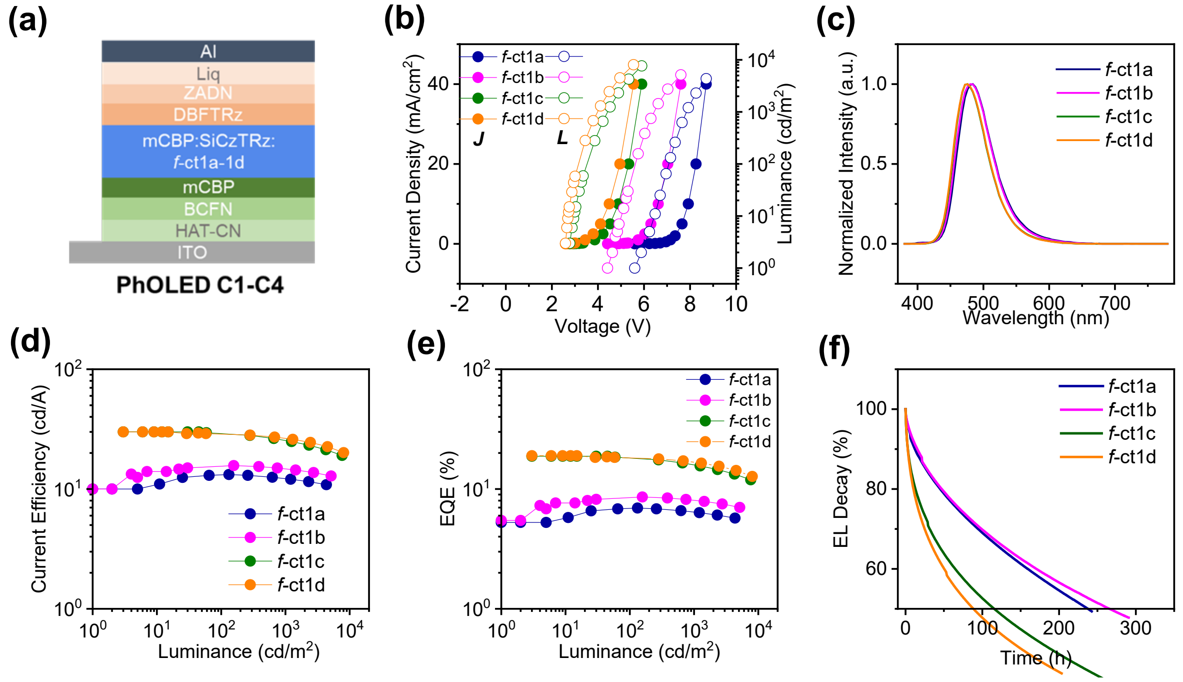

Supplementary Fig. 25. (a) Schematic device structure; (b) Current density-voltage-luminance (J-V-L) characteristics; (c) EL spectra; (d) CE-luminance characteristics; (e) EQE-luminance characteristics; (f) Normalized luminance with a L_0 = 500 cd m^{-2} as a function of operational time. All doping conc. of Ir(III) complexes is maintained at 21 wt%.

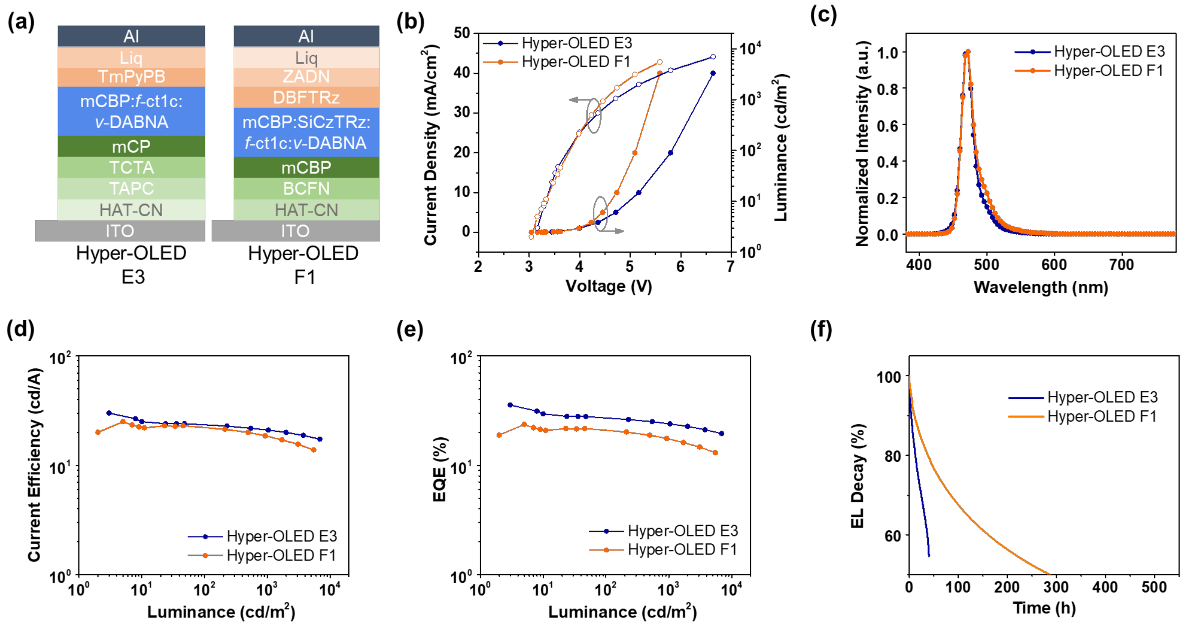

Supplementary Fig. 26. Sensitized OLEDs based on mCBP and mCBP:SiCzTRz as the host. (a) Schematic device structures; (b) Current density-voltage-luminance (J-V-L) characteristics; (c) EL spectra, (d) CE-luminance characteristics; (e) EQE-luminance characteristics; (f) Normalized luminance with a L_0 = 500 cd m^{-2} as a function of operational time. All doping conc. of Ir(III) complexes is maintained at 21 wt%.

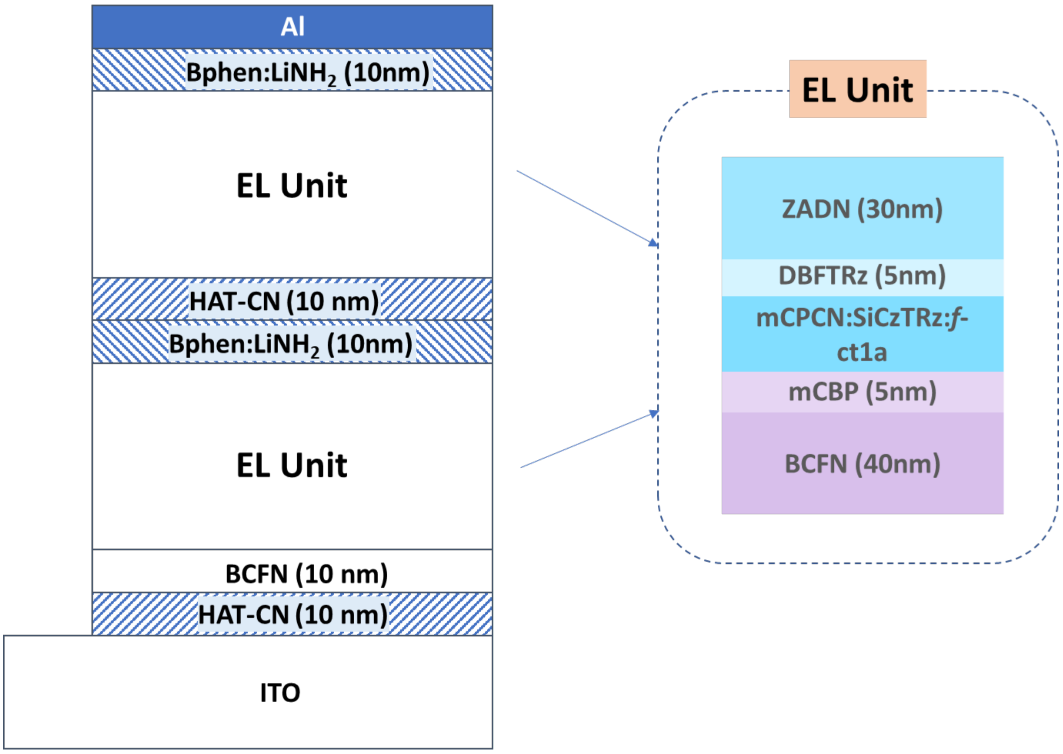

Supplementary Fig. 27. Schematic device structure of tandem OLED devices.

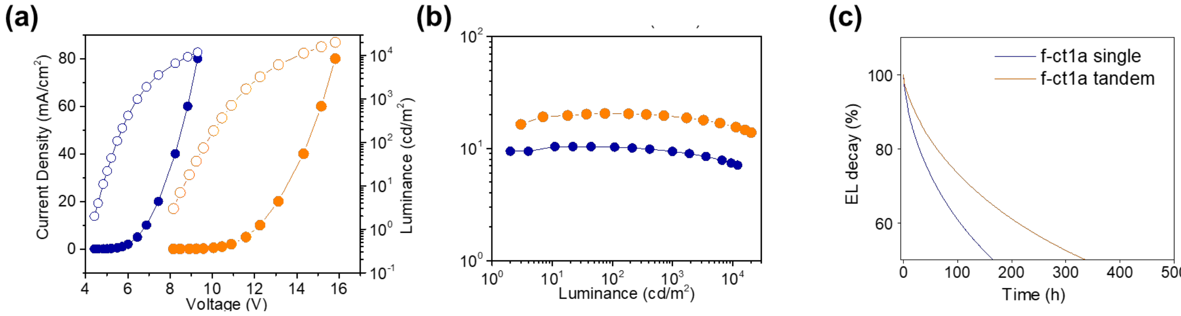

Supplementary Fig. 28. (a) Current density-voltage-luminance (J-V-L) characteristics of single and tandem devices using f-ct1a as the emitter and mCBP:SiCzTRz as the co-host; (b) EQE-luminance characteristics; (c) Normalized luminance with a L_0 = 500 cd m^{-2} as a function of operational time. All doping conc. of Ir(III) complexes is maintained at 21 wt%.

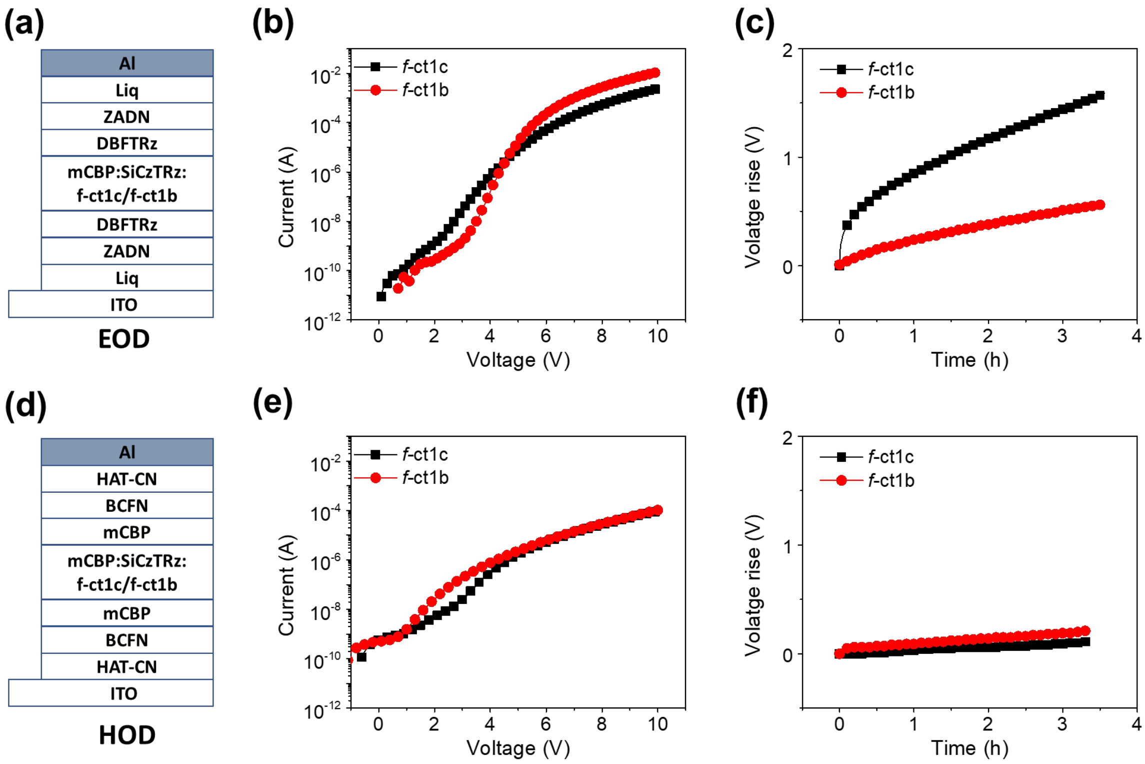

Supplementary Fig. 29. (a) Device structure of electron-only devices using f-ct1b and f-ct1c as the emitter; (b) Current-voltage characteristics; (c) Voltage rise as a function of operational time; (d) Device structure of hole-only devices using emitters f-ct1b and f-ct1c; (e) Current-voltage characteristics; (f) Voltage rise as a function of operational time.

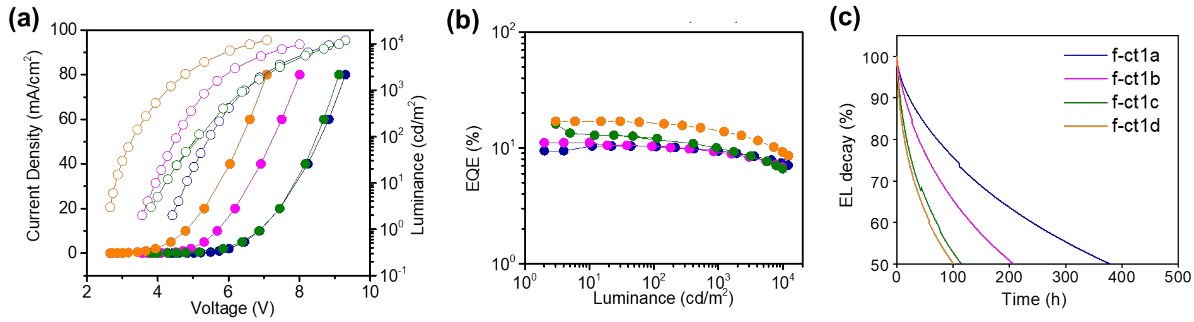

Supplementary Fig. 30. (a) Current density-voltage-luminance (J-V-L) characteristics of devices using emitters f-ct1a – d and mCPCN:SiCzTRz as the co-host; (b) EQE-luminance characteristics; (c) Normalized luminance with a L_0 = 500 cd m^{-2} as a function of operational time. All doping conc. of Ir(III) complexes is maintained at 21 wt%.

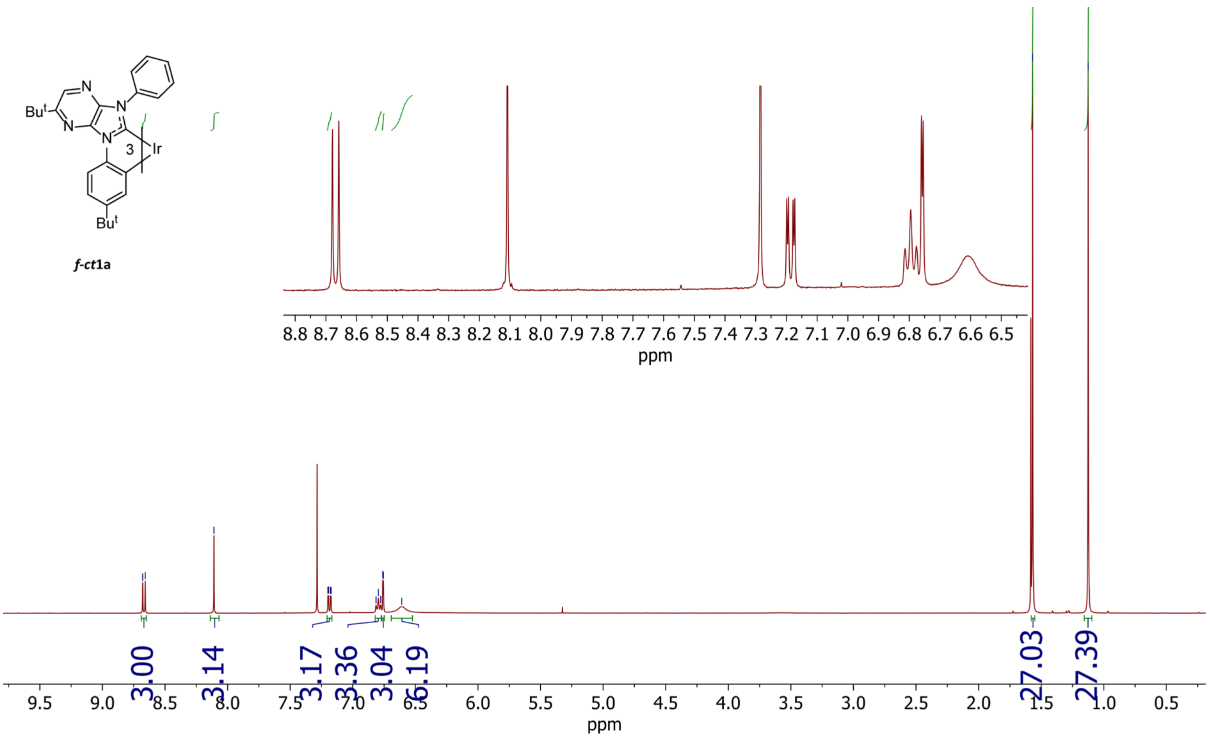

Supplementary Fig. 31. ^{1}H NMR (400 MHz) spectrum of f-ct1a in $CDCl_3$ solution at RT.

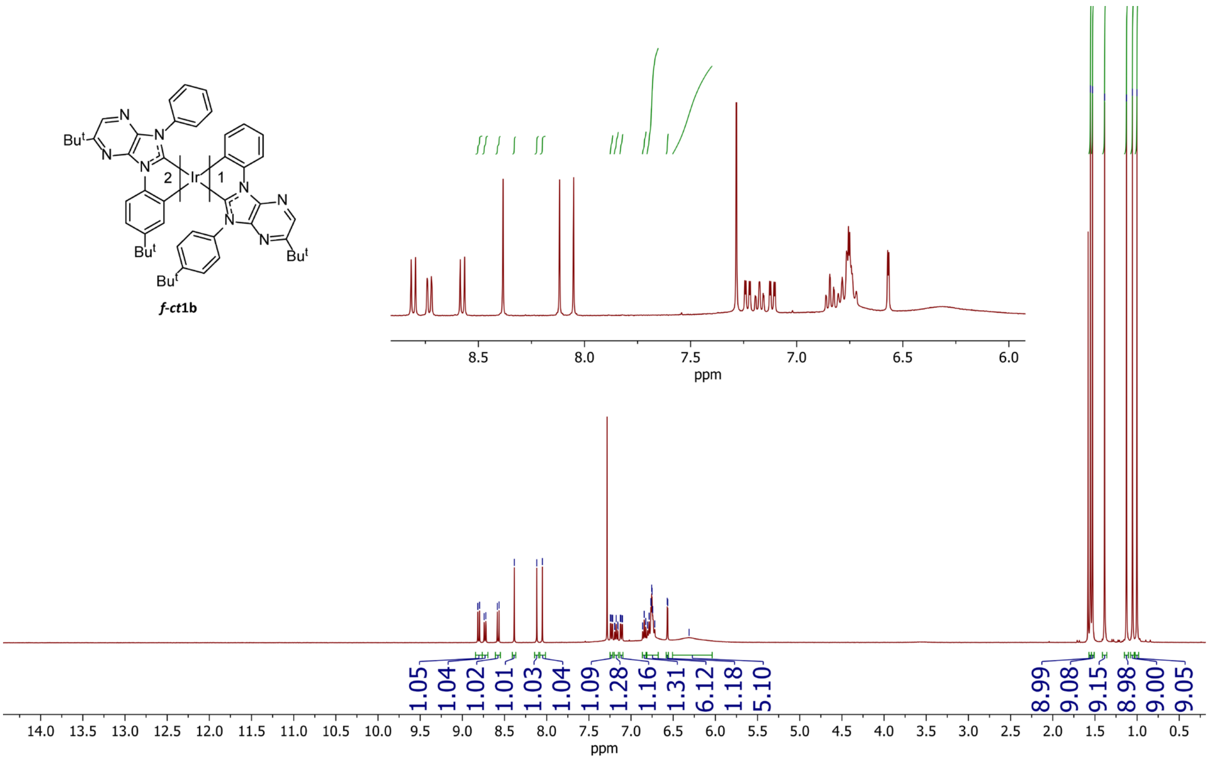

Supplementary Fig. 32. ^{1}H NMR (400 MHz) spectrum of f-ct1b in $CDCl_3$ solution at RT.

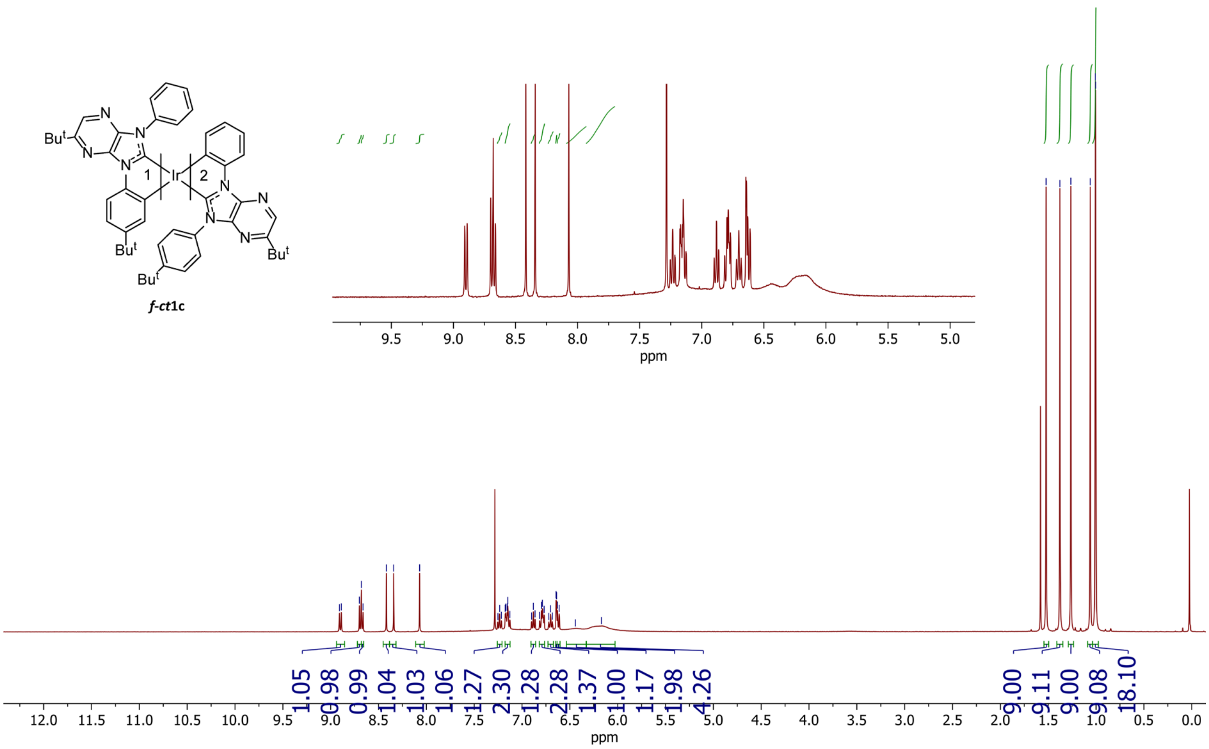

Supplementary Fig. 33. ^{1}H NMR (400 MHz) spectrum of f-ct1c in $CDCl_3$ solution at RT.

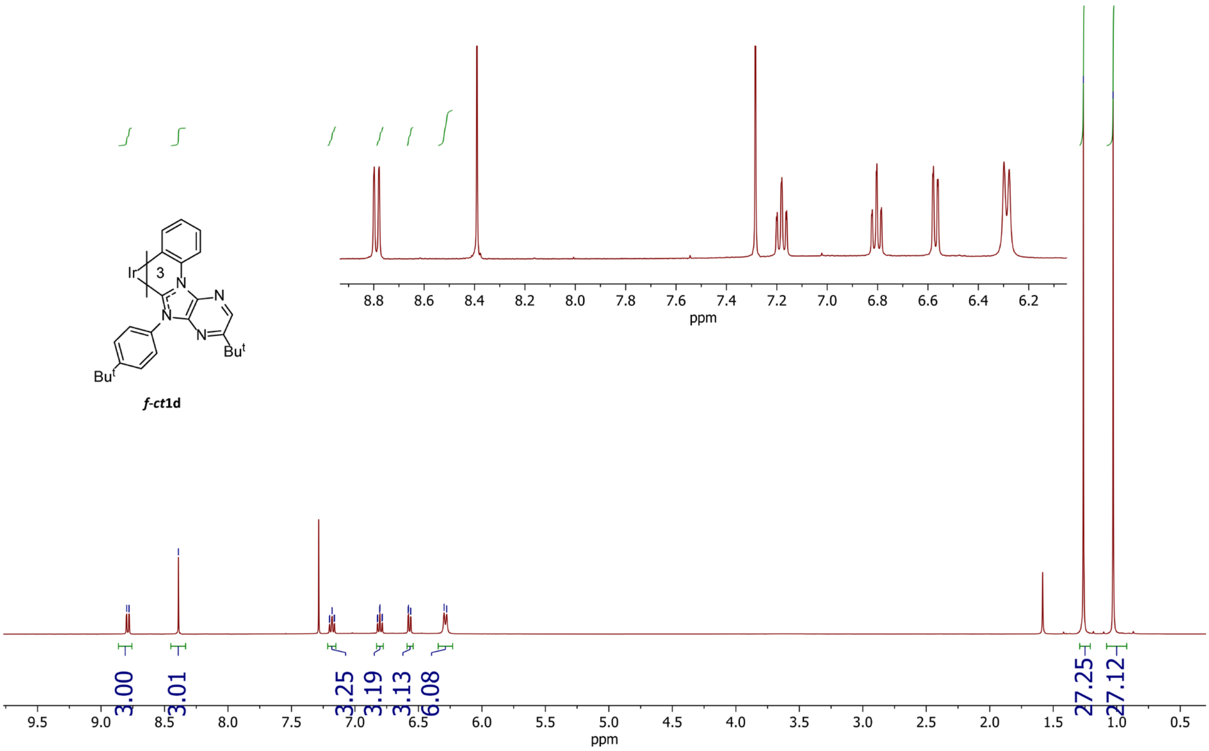

Supplementary Fig. 34. ^{1}H NMR (400 MHz) spectrum of f-ct1d in $CDCl_3$ solution at RT.

Supplementary Table 1. The calculation results[a] for the excitation energy (ϵ), oscillator strength (*f*), main orbital contributions of the lowest singlet (S_1) and triplet (T_1) excited states, and the assignment of the charge character of T_1 state for the studied Ir(III) complexes at their geometries optimized for the ground state.

	State	ϵ (nm/eV)	*f*	Orbital contribution (> 20%)	Assignment
f-ct1a	T_1	453.6/2.73	0	HOMO → LUMO (62%)	MLCT (20.7%), LC, ILCT, LLCT
	S_1	420.2/2.95	0.0757	HOMO → LUMO+1 (94%)	
f-ct1b	T_1	458.2/2.71	0	HOMO → LUMO (76%)	MLCT (21.5%), LC, ILCT, LLCT
	S_1	424.5/2.92	0.0625	HOMO → LUMO (75%)	
f-ct1c	T_1	451.9/2.74	0	HOMO → LUMO (67%)	MLCT (21.5%), LC, ILCT, LLCT
	S_1	417.3/2.97	0.0655	HOMO → LUMO (51%)	
f-ct1d	T_1	451.6/2.75	0	HOMO → LUMO (70%)	MLCT (21.2%), LC, ILCT
	S_1	417.0/2.97	0.0809	HOMO → LUMO+1 (95%)	

[a] The results were calculated by TD-DFT using B3LYP functional with PCM for modeling the toluene solvent (see computational details for more information).
[b] The percentage of MLCT character of T_1 state was calculated as the change in the metal contribution from the occupied to the virtual NTOs multiplied with the eigenvalues of the corresponding NTOs (*cf*. Figure 1).

Supplementary Table 2. The relativistic calculation results for the excitation energy (ϵ) and corresponding oscillator strength (f), calculated radiative decay rate (k_r), and the assignment of charge characters of the lowest triplet (T_1) excited states for studied Ir(III) complexes at their geometries optimized for the ground state.

	ϵ [a]	f (10^{-4}) [a]	k_r (10^5 s^{-1}) [b]
f-ct1a	459 nm / 2.72 eV	3.0	0.9
f-ct1b	462 nm / 2.68 eV	3.3	1.0
f-ct1c	452 nm / 2.74 eV	3.7	1.2
f-ct1d	451 nm / 2.75 eV	3.3	1.1

[a] The calculated ϵ, f, and k_r of T_1 state are averaged from the three substates of T_1 state, where spin-mixed excitations were calculated by relativistic calculations with spin–orbit coupling (SOC) added perturbatively to one-component TD-DFT, and with COSMO for

modeling the toluene solvent (see additional computational details in the Supporting Information for more information).

[b] Note that the calculated k_r (at 0 K) is systematically smaller than the experimental k_r (at RT), same as that found in other families of Ir(III) complexes. The former is intrinsic and independent from the non-radiative decay pathways, whereas the latter is highly dependent on the non-radiative decay pathways. The uncertainty of applied computational model and methods is also one of possible sources of deviation between calculated and experimental k_r. In general, the trend of calculated and experimental k_r is consistent. Also, the trend of calculated k_r for four Ir(III) complexes (*cf*. **Supplementary Table 2**) is similar to that determined by experiments (*cf*. **Table 1**), that is, f-ct1c achieved fastest k_r among four complexes.

Supplementary Table 3. Summary of decomposition temperature (T_d), voltammetric data and calculated energy gaps.

	T_d (°C) [a]	$E^{ox}_{½}$ (V) (ΔE_p) [b]	HOMO (eV) [c]	energy gap (eV) [d]	LUMO (eV) [e]
f-ct1a	372	0.59 (0.08)	−5.39	2.84	−2.55
f-ct1b	359	0.62 (0.07)	−5.42	2.85	−2.57
f-ct1c	376	0.67 (0.08)	−5.47	2.87	−2.60
f-ct1d	401	0.69 (0.09)	−5.49	2.89	−2.60

[a] Temperature with a weight loss of 5%. [b] These data were measured in a 0.1 M acetonitrile solution of $TBAPF_6$. $\mathbf{E_{ox}}$ refers to $[(E_{pa} + E_{pc})/2]$, where E_{pa} and E_{pc} are the anodic and cathodic peak potentials referenced to the Fc/Fc^+ couple; [c] HOMO = $-(E^{ox}_{½} + 4.8)$; [d] energy gap = 1240 / [PL_{onset} (nm)]; [e] LUMO = HOMO + energy gap.

Supplementary Table 4. EQE-CIE$_y$ relationship of recently reported ν-DABNA-based hyper-OLED devices with different sensitizers.

Symbols	V_{on} [V]	EQE_{max}	λ_{EL} [nm]	CIE (x, y)	FWHM [nm]	Sensitizer	citation
■	4.0	16.6%	472	0.14, 0.18	23	Au-1	[14]
●	–	23.4%	469	0.13, 0.12	18	Complex 5	[15]
▲	3.0	27%	470	0.15, 0.20	18	HDT-1	[16]
▼	3.1	38.8%	473	0.12, 0.15	19	DBA-BFICz	[17]
◆	3.0	33.5%	473	0.12, 0.18	19	PCzTRz	[18]
◀	3.0	32.2%	473	0.111, 0.141	20	PtON7-dtb	[19]
▶	3.4	34.4%	469	0.12, 0.11	18	DOBNA-OAr	[20]
⬢	3.0	27.3%	470	0.132, 0.162	20	CN-Ir	[21]
★	–	34.7%	471	0.13, 0.15	19	TSF-dCz	[22]
⬟	3.5	22%	472	0.120, 0.155	21	m-2-tBu	[23]
●	3.1	35.5%	472	0.119, 0.107	18	***f*-ct1c**	this work

Supplementary References

1 te Velde, G. *et al.* Chemistry with ADF. *J. Comput. Chem.* **22**, 931-967 (2001).

2 Van Lenthe, E. & Baerends, E. J. Optimized Slater-type basis sets for the elements 1–118. *J. Comput. Chem.* **24**, 1142-1156 (2003).

3 Chong, D. P. Augmenting basis set for time-dependent density functional theory calculation of excitation energies: Slater-type orbitals for hydrogen to krypton. *Mol. Phys.* **103**, 749-761 (2005).

4 Klamt, A. & Schuurmann, G. COSMO: a new approach to dielectric screening in solvents with explicit expressions for the screening energy and its gradient. *J. Chem. Soc., Perkin Trans. 2*, 799-805 (1993).

5 Wang, F. & Ziegler, T. A Simplified Relativistic Time-Dependent Density-Functional Theory Formalism for the Calculations of Excitation Energies Including Spin-Orbit Coupling Effect. *J. Chem. Phys.* **123**, 154102 (2005).

6 Casida, M. E. Time-Dependent Density-Functional Theory for Molecules and Molecular Solids. *J. Mol. Struct. THEOCHEM* **914**, 3-18 (2009).

7 Chang, C., Pelissier, M. & Durand, P. Regular Two-Component Pauli-Like Effective Hamiltonians in Dirac Theory. *Phys. Scr.* **34**, 394-404 (1986).

8 van Lenthe, E., Baerends, E. J. & Snijders, J. G. Relativistic total energy using regular approximations. *J. Chem. Phys.* **101**, 9783-9792 (1994).

9 Strickler, S. J. & Berg, R. A. Relationship between Absorption Intensity and Fluorescence Lifetime of Molecules. *J. Chem. Phys.* **37**, 814-822 (1962).

10 Nozaki, K. Theoretical Studies on Photophysical Properties and Mechanism of Phosphorescence in [fac-Ir(2-phenylpyridine)3]. *J. Chin. Chem. Soc.* **53**, 101-112 (2006).

11 Zhou, X. & Powell, B. J. Nonradiative Decay and Stability of N-Heterocyclic Carbene Iridium(III) Complexes. *Inorg. Chem.* **57**, 8881-8889 (2018).

12 Powell, B. J. Theories of phosphorescence in organo-transition metal complexes – From relativistic effects to simple models and design principles for organic light-emitting diodes. *Coord. Chem. Rev.* **295**, 46-79 (2015).

13 Powell, B. J. Conservation laws, radiative decay rates and excited state localization in organometallic complexes with strong spin-orbit coupling. *Sci. Rep.* **5**, 10815 (2015).

14 Zhou, D., Wu, S., Cheng, G. & Che, C.-M. A gold(iii)–TADF emitter as a sensitizer for high-color-purity and efficient deep-blue solution-processed OLEDs. *J. Mater. Chem. C* **10**, 4590-4596 (2022).

15 Lo, K.-W., Tong, G. S. M., Cheng, G., Low, K.-H. & Che, C.-M. Dinuclear Pt^{II} Complexes with Strong Blue Phosphorescence for Operationally Stable Organic Light-Emitting Diodes with EQE up to 23 % at 1000 cd m−2. *Angew. Chem. Int. Ed.* **61**, e202115515 (2022).

16 Chan, C.-Y. *et al.* Stable pure-blue hyperfluorescence organic light-emitting diodes with high-efficiency and narrow emission. *Nat. Photonics* **15**, 203–207 (2021).
17 Braveenth, R. *et al.* Achieving Narrow FWHM and High EQE Over 38% in Blue OLEDs Using Rigid Heteroatom-Based Deep Blue TADF Sensitized Host. *Adv. Funct. Mater.* **31**, 2105805 (2021).
18 Jeon, S. O. *et al.* High-efficiency, long-lifetime deep-blue organic light-emitting diodes. *Nat. Photonics* **15**, 208-215 (2021).
19 Nam, S. *et al.* Improved Efficiency and Lifetime of Deep-Blue Hyperfluorescent Organic Light-Emitting Diode using Pt(II) Complex as Phosphorescent Sensitizer. *Adv. Sci.* **8**, 2100586 (2021).
20 Kondo, Y. *et al.* Narrowband deep-blue organic light-emitting diode featuring an organoboron-based emitter. *Nat. Photonics* **13**, 678-682 (2019).
21 Chung, W. J. *et al.* Over 30 000 h Device Lifetime in Deep Blue Organic Light-Emitting Diodes with y Color Coordinate of 0.086 and Current Efficiency of 37.0 cd A^{-1}. *Adv. Opt. Mater.* **9**, 2100203 (2021).
22 Huang, T., Wang, Q., Meng, G., Duan, L. & Zhang, D. Accelerating Radiative Decay in Blue Through-Space Charge Transfer Emitters by Minimizing the Face-to-Face Donor–Acceptor Distances. *Angew. Chem. Int. Ed.* **61**, e202200059 (2022).
23 Jin, J. *et al.* Iridium(III) Phosphors–Bearing Functional 9-Phenyl-7,9-dihydro-8H-purin-8-ylidene Chelates and Blue Hyperphosphorescent OLED Devices. *Adv. Photon. Res.* **3**, 2100381 (2022).
