# Supplementary material for: Electroluminescence and hyperphosphorescence from stable blue Ir(III) carbene complexes with suppressed efficiency roll-off
Source: Nat Commun. 2023 Oct 12;14:6419. doi: 10.1038/s41467-023-42090-z (PMC10570383; doi:10.1038/s41467-023-42090-z)
Supplement: Supplementary file 3 — Description of Additional Supplementary Files [file 41467_2023_42090_MOESM3_ESM.pdf]

File name: Supplementary Data 1

Description: f-ct1a_checkcif

File name: Supplementary Data 2

Description: f-ct1b_checkcif

File name: Supplementary Data 3

Description: f-ct1c_checkcif

File name: Supplementary Data 4

Description: f-ct1d_checkcif

File name: Supplementary Data 5

Description: Cartesian coordinates of the optimized geometries
