# Supplementary material for: Electroluminescence and hyperphosphorescence from stable blue Ir(III) carbene complexes with suppressed efficiency roll-off
Source: Nat Commun. 2023 Oct 12;14:6419. doi: 10.1038/s41467-023-42090-z (PMC10570383; doi:10.1038/s41467-023-42090-z)

checkCIF/PLATON report

Structure factors have been supplied for datablock(s) 1_a_sq

THIS REPORT IS FOR GUIDANCE ONLY. IF USED AS PART OF A REVIEW PROCEDURE FOR PUBLICATION, IT SHOULD NOT REPLACE THE EXPERTISE OF AN EXPERIENCED CRYSTALLOGRAPHIC REFEREE.

No syntax errors found. CIF dictionary Interpreting this report

Datablock: 1_a_sq

```
Bond precision:   C-C = 0.0042 A          Wavelength=1.54178

Cell:                a=38.484(8)       b=38.484(8)       c=24.409(5)
                     alpha=90          beta=90           gamma=120
Temperature:         193 K
```

	Calculated	Reported
Volume	31307(15)	31307(15)
Space group	R -3	R -3 :H
Hall group	-R 3	-R 3
Moiety formula	C75 H81 Ir N12 [+ solvent]	?
Sum formula	C75 H81 Ir N12 [+ solvent]	C75 H81 Ir N12
Mr	1342.74	1342.71
Dx,g cm-3	1.282	1.282
Z	18	18
Mu (mm-1)	4.098	4.098
F000	12456.0	12456.0
F000'	12398.82	
h,k,lmax	48,48,30	47,48,30
Nref	14276	14251
Tmin,Tmax	0.582,0.849	0.440,0.754
Tmin'	0.403	

```
Correction method= # Reported T Limits: Tmin=0.440 Tmax=0.754
AbsCorr = MULTI-SCAN

Data completeness= 0.998           Theta(max)= 74.644

R(reflections)= 0.0276( 13297)                    wR2(reflections)=
                                                  0.0739( 14251)

S = 1.004                    Npar= 850
```

The following ALERTS were generated. Each ALERT has the format
test-name_ALERT_alert-type_alert-level.
Click on the hyperlinks for more details of the test.

Alert level C

```
PLAT048_ALERT_1_C MoietyFormula Not Given (or Incomplete) ........     Please Check
PLAT213_ALERT_2_C Atom C48             has ADP max/min Ratio .....        3.2 prolat
PLAT220_ALERT_2_C NonSolvent   Resd 1  C   Ueq(max)/Ueq(min) Range        5.1 Ratio
PLAT222_ALERT_3_C NonSolvent Resd 1  H   Uiso(max)/Uiso(min) Range        5.5 Ratio
PLAT242_ALERT_2_C Low    'MainMol' Ueq as Compared to Neighbors of        C18 Check
PLAT242_ALERT_2_C Low    'MainMol' Ueq as Compared to Neighbors of        C43 Check
PLAT242_ALERT_2_C Low    'MainMol' Ueq as Compared to Neighbors of        C68 Check
PLAT911_ALERT_3_C Missing FCF Refl Between Thmin & STh/L=    0.600          8 Report
```

Alert level G

```
PLAT002_ALERT_2_G Number of Distance or Angle Restraints on AtSite          8 Note
PLAT003_ALERT_2_G Number of Uiso or Uij Restrained non-H Atoms ...          8 Report
PLAT083_ALERT_2_G SHELXL Second Parameter in WGHT  Unusually Large      25.13 Why ?
PLAT172_ALERT_4_G The CIF-Embedded .res File Contains DFIX Records          2 Report
PLAT177_ALERT_4_G The CIF-Embedded .res File Contains DELU Records          1 Report
PLAT178_ALERT_4_G The CIF-Embedded .res File Contains SIMU Records          1 Report
PLAT186_ALERT_4_G The CIF-Embedded .res File Contains ISOR Records          1 Report
PLAT232_ALERT_2_G Hirshfeld Test Diff (M-X)  Ir1      --C26      .        6.3 s.u.
PLAT232_ALERT_2_G Hirshfeld Test Diff (M-X)  Ir1      --C51      .        5.4 s.u.
PLAT300_ALERT_4_G Atom Site Occupancy of C47        Constrained at        0.6 Check
PLAT300_ALERT_4_G Atom Site Occupancy of C48        Constrained at        0.6 Check
PLAT300_ALERT_4_G Atom Site Occupancy of C49        Constrained at        0.6 Check
PLAT300_ALERT_4_G Atom Site Occupancy of C50        Constrained at        0.6 Check
PLAT300_ALERT_4_G Atom Site Occupancy of C47A       Constrained at        0.4 Check
PLAT300_ALERT_4_G Atom Site Occupancy of C48A       Constrained at        0.4 Check
PLAT300_ALERT_4_G Atom Site Occupancy of C49A       Constrained at        0.4 Check
PLAT300_ALERT_4_G Atom Site Occupancy of C50A       Constrained at        0.4 Check
PLAT300_ALERT_4_G Atom Site Occupancy of H48A       Constrained at        0.6 Check
PLAT300_ALERT_4_G Atom Site Occupancy of H48B       Constrained at        0.6 Check
PLAT300_ALERT_4_G Atom Site Occupancy of H48C       Constrained at        0.6 Check
PLAT300_ALERT_4_G Atom Site Occupancy of H49A       Constrained at        0.6 Check
PLAT300_ALERT_4_G Atom Site Occupancy of H49B       Constrained at        0.6 Check
PLAT300_ALERT_4_G Atom Site Occupancy of H49C       Constrained at        0.6 Check
PLAT300_ALERT_4_G Atom Site Occupancy of H50A       Constrained at        0.6 Check
PLAT300_ALERT_4_G Atom Site Occupancy of H50B       Constrained at        0.6 Check
PLAT300_ALERT_4_G Atom Site Occupancy of H50C       Constrained at        0.6 Check
PLAT300_ALERT_4_G Atom Site Occupancy of H48D       Constrained at        0.4 Check
PLAT300_ALERT_4_G Atom Site Occupancy of H48E       Constrained at        0.4 Check
PLAT300_ALERT_4_G Atom Site Occupancy of H48F       Constrained at        0.4 Check
PLAT300_ALERT_4_G Atom Site Occupancy of H49D       Constrained at        0.4 Check
PLAT300_ALERT_4_G Atom Site Occupancy of H49E       Constrained at        0.4 Check
PLAT300_ALERT_4_G Atom Site Occupancy of H49F       Constrained at        0.4 Check
PLAT300_ALERT_4_G Atom Site Occupancy of H50D       Constrained at        0.4 Check
PLAT300_ALERT_4_G Atom Site Occupancy of H50E       Constrained at        0.4 Check
PLAT300_ALERT_4_G Atom Site Occupancy of H50F       Constrained at        0.4 Check
PLAT301_ALERT_3_G Main Residue  Disorder ..............(Resd  1  )         5% Note
PLAT412_ALERT_2_G Short Intra XH3 .. XHn     H35      ..H50B     .       2.09 Ang.
                                                    x,y,z  =      1_555 Check
PLAT605_ALERT_4_G Largest Solvent Accessible VOID in the Structure        484 A**3
```

```
PLAT860_ALERT_3_G Number of Least-Squares Restraints ............        180 Note
PLAT869_ALERT_4_G ALERTS Related to the Use of SQUEEZE  Suppressed         ! Info
PLAT883_ALERT_1_G No Info/Value for _atom_sites_solution_primary .     Please Do !
PLAT910_ALERT_3_G Missing # of FCF Reflection(s) Below Theta(Min).         1 Note
PLAT912_ALERT_4_G Missing # of FCF Reflections Above STh/L=  0.600        17 Note
PLAT933_ALERT_2_G Number of HKL-OMIT Records in Embedded .res File         1 Note
PLAT965_ALERT_2_G The SHELXL WEIGHT Optimisation has not Converged     Please Check
PLAT978_ALERT_2_G Number C-C Bonds with Positive Residual Density.         1 Info
```

```
  0 ALERT level A = Most likely a serious problem - resolve or explain
  0 ALERT level B = A potentially serious problem, consider carefully
  8 ALERT level C = Check. Ensure it is not caused by an omission or oversight
 46 ALERT level G = General information/check it is not something unexpected

  2 ALERT type 1 CIF construction/syntax error, inconsistent or missing data
 14 ALERT type 2 Indicator that the structure model may be wrong or deficient
  5 ALERT type 3 Indicator that the structure quality may be low
 33 ALERT type 4 Improvement, methodology, query or suggestion
  0 ALERT type 5 Informative message, check
```

Publication of your CIF

A full structural check has been run on your CIF. This includes checks on:

- CIF syntax and construction
- Cell and geometry details
- Space-group symmetry
- Anisotropic displacement parameters

Structure-factor checking is currently being tested on articles submitted to *Acta Crystallographica Section C* and *Acta Crystallographica Section E*. These tests may be carried out with a local version of PLATON or the trial service here.

These full checks give an indication of potential problems with your CIF. Please note that if you intend to submit your CIF for publication in Acta Crystallographica Section C or E or IUCrData, you must make sure that full publication checks are run on the final version of the CIF prior to submission.

If you intend to submit to another section of Acta Crystallographica, Journal of Applied Crystallography or Journal of Synchrotron Radiation, you should make sure that at least basic structural checks are run on the final version of your CIF prior to submission.

To submit your CIF for publication in an IUCr journal click here.

PLATON version of 19/02/2022; check.def file version of 19/01/2022

Datablock 1_a_sq - ellipsoid plot

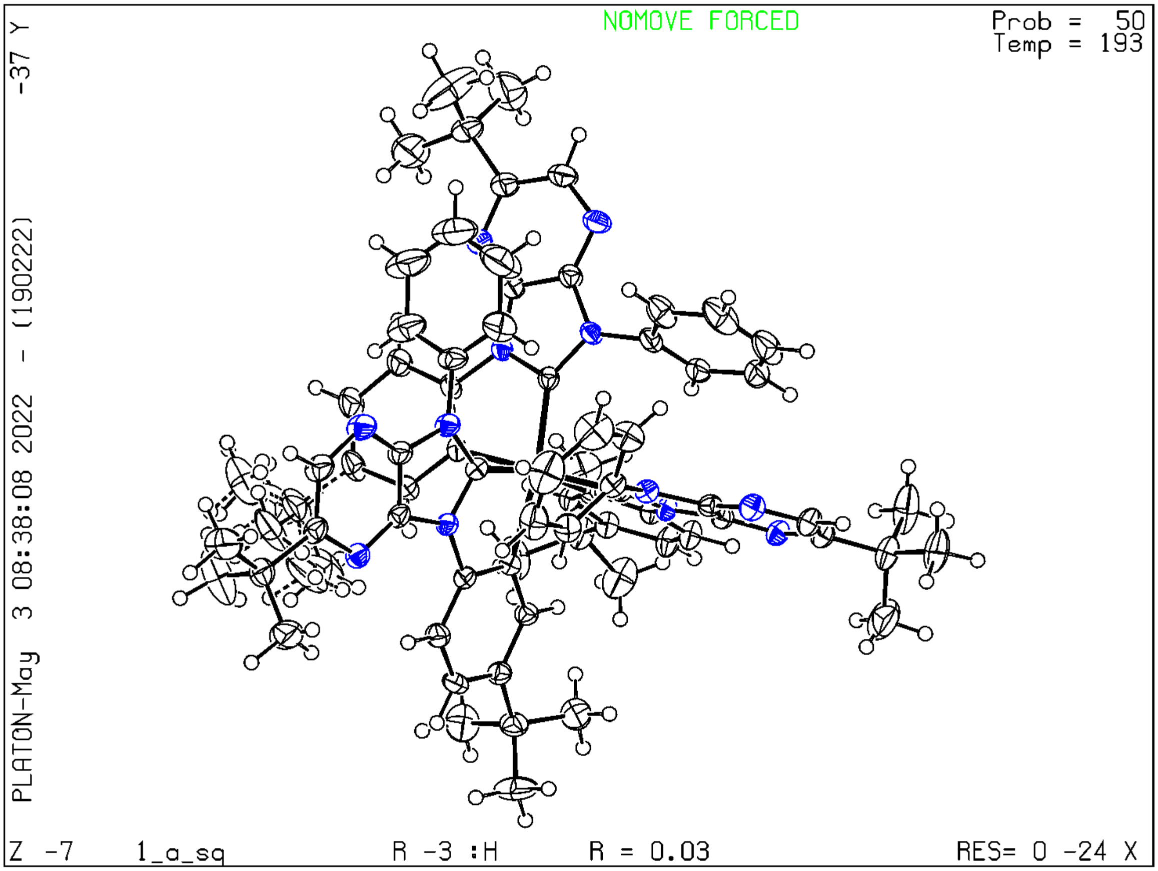

Supplement: Supplementary file 4 — Supplementary Data 1 [file 41467_2023_42090_MOESM4_ESM.pdf]
