# Supplementary material for: Electroluminescence and hyperphosphorescence from stable blue Ir(III) carbene complexes with suppressed efficiency roll-off
Source: Nat Commun. 2023 Oct 12;14:6419. doi: 10.1038/s41467-023-42090-z (PMC10570383; doi:10.1038/s41467-023-42090-z)

checkCIF/PLATON report

Structure factors have been supplied for datablock(s) 1_a

THIS REPORT IS FOR GUIDANCE ONLY. IF USED AS PART OF A REVIEW PROCEDURE FOR PUBLICATION, IT SHOULD NOT REPLACE THE EXPERTISE OF AN EXPERIENCED CRYSTALLOGRAPHIC REFEREE.

No syntax errors found. CIF dictionary Interpreting this report

Datablock: 1_a

Bond precision:	C-C = 0.0069 A		Wavelength=0.71073
Cell:	a=13.9005(4)	b=26.4544(7)	c=19.8474(5)
	alpha=90	beta=100.541(1)	gamma=90
Temperature:	193 K		

	Calculated	Reported
Volume	7175.3(3)	7175.3(3)
Space group	P 21/c	P 21/c
Hall group	-P 2ybc	-P 2ybc
Moiety formula	2(C75 H81 Ir N12), C H2 Cl2	?
Sum formula	C151 H164 Cl2 Ir2 N24	C75.50 H82 Cl Ir N12
Mr	2770.41	1385.18
Dx,g cm-3	1.282	1.282
Z	2	4
Mu (mm-1)	1.947	1.947
F000	2852.0	2852.0
F000'	2847.59	
h,k,lmax	17,33,24	17,33,24
Nref	14712	14684
Tmin,Tmax	0.728,0.943	0.572,0.745
Tmin'	0.491	

Correction method= # Reported T Limits: Tmin=0.572 Tmax=0.745
AbsCorr = MULTI-SCAN

Data completeness= 0.998 Theta(max)= 26.395

R(reflections)= 0.0381(12203) wR2(reflections)= 0.1368(14684)

S = 1.053 Npar= 922

The following ALERTS were generated. Each ALERT has the format
test-name_ALERT_alert-type_alert-level.
Click on the hyperlinks for more details of the test.

Alert level C

```
PLAT048_ALERT_1_C MoietyFormula Not Given (or Incomplete) ........     Please Check
PLAT085_ALERT_2_C SHELXL Default Weighting Scheme is not Optimized     Please Check
PLAT213_ALERT_2_C Atom C24             has ADP max/min Ratio .....        3.3 prolat
PLAT213_ALERT_2_C Atom C25             has ADP max/min Ratio .....        3.5 prolat
PLAT220_ALERT_2_C NonSolvent   Resd 1  C   Ueq(max)/Ueq(min) Range        5.3 Ratio
PLAT222_ALERT_3_C NonSolvent Resd 1  H   Uiso(max)/Uiso(min) Range        5.6 Ratio
PLAT234_ALERT_4_C Large Hirshfeld Difference C68      --C70      .       0.17 Ang.
PLAT242_ALERT_2_C Low    'MainMol' Ueq as Compared to Neighbors of        C22 Check
PLAT242_ALERT_2_C Low    'MainMol' Ueq as Compared to Neighbors of        C47 Check
PLAT242_ALERT_2_C Low    'MainMol' Ueq as Compared to Neighbors of        C68 Check
PLAT601_ALERT_2_C Unit Cell Contains Solvent Accessible VOIDS of .         58 Ang**3
PLAT910_ALERT_3_C Missing # of FCF Reflection(s) Below Theta(Min).          9 Note
PLAT911_ALERT_3_C Missing FCF Refl Between Thmin & STh/L=    0.600         12 Report
PLAT971_ALERT_2_C Check Calcd Resid. Dens.  3.12Ang From Cl2             1.57 eA-3
PLAT971_ALERT_2_C Check Calcd Resid. Dens.  2.46Ang From Cl2             1.53 eA-3
PLAT977_ALERT_2_C Check Negative Difference Density on H75F      .      -0.38 eA-3
PLAT977_ALERT_2_C Check Negative Difference Density on H75B      .      -0.34 eA-3
```

Alert level G

```
PLAT002_ALERT_2_G Number of Distance or Angle Restraints on AtSite         19 Note
PLAT003_ALERT_2_G Number of Uiso or Uij Restrained non-H Atoms ...         24 Report
PLAT045_ALERT_1_G Calculated and Reported Z Differ by a Factor ...         1. Check
PLAT168_ALERT_4_G The CIF-Embedded .res File Contains EXYZ Records          2 Report
PLAT171_ALERT_4_G The CIF-Embedded .res File Contains EADP Records          2 Report
PLAT172_ALERT_4_G The CIF-Embedded .res File Contains DFIX Records          5 Report
PLAT173_ALERT_4_G The CIF-Embedded .res File Contains DANG Records          1 Report
PLAT177_ALERT_4_G The CIF-Embedded .res File Contains DELU Records          3 Report
PLAT178_ALERT_4_G The CIF-Embedded .res File Contains SIMU Records          3 Report
PLAT186_ALERT_4_G The CIF-Embedded .res File Contains ISOR Records          3 Report
PLAT300_ALERT_4_G Atom Site Occupancy of C18        Constrained at       0.65 Check
PLAT300_ALERT_4_G Atom Site Occupancy of C19        Constrained at       0.65 Check
PLAT300_ALERT_4_G Atom Site Occupancy of C20        Constrained at       0.65 Check
PLAT300_ALERT_4_G Atom Site Occupancy of C21        Constrained at       0.65 Check
PLAT300_ALERT_4_G Atom Site Occupancy of C43        Constrained at        0.7 Check
PLAT300_ALERT_4_G Atom Site Occupancy of C44        Constrained at        0.7 Check
PLAT300_ALERT_4_G Atom Site Occupancy of C45        Constrained at        0.7 Check
PLAT300_ALERT_4_G Atom Site Occupancy of C46        Constrained at        0.7 Check
PLAT300_ALERT_4_G Atom Site Occupancy of C72A       Constrained at        0.6 Check
PLAT300_ALERT_4_G Atom Site Occupancy of C73A       Constrained at        0.6 Check
PLAT300_ALERT_4_G Atom Site Occupancy of C74A       Constrained at        0.6 Check
PLAT300_ALERT_4_G Atom Site Occupancy of C75A       Constrained at        0.6 Check
PLAT300_ALERT_4_G Atom Site Occupancy of C18A       Constrained at       0.35 Check
PLAT300_ALERT_4_G Atom Site Occupancy of C19A       Constrained at       0.35 Check
PLAT300_ALERT_4_G Atom Site Occupancy of C20A       Constrained at       0.35 Check
PLAT300_ALERT_4_G Atom Site Occupancy of C21A       Constrained at       0.35 Check
PLAT300_ALERT_4_G Atom Site Occupancy of C43A       Constrained at        0.3 Check
PLAT300_ALERT_4_G Atom Site Occupancy of C44A       Constrained at        0.3 Check
PLAT300_ALERT_4_G Atom Site Occupancy of C45A       Constrained at        0.3 Check
PLAT300_ALERT_4_G Atom Site Occupancy of C46A       Constrained at        0.3 Check
```

```
PLAT300_ALERT_4_G Atom Site Occupancy of C72        Constrained at        0.4 Check
PLAT300_ALERT_4_G Atom Site Occupancy of C73        Constrained at        0.4 Check
PLAT300_ALERT_4_G Atom Site Occupancy of C74        Constrained at        0.4 Check
PLAT300_ALERT_4_G Atom Site Occupancy of C75        Constrained at        0.4 Check
PLAT300_ALERT_4_G Atom Site Occupancy of H19A       Constrained at       0.65 Check
PLAT300_ALERT_4_G Atom Site Occupancy of H19B       Constrained at       0.65 Check
PLAT300_ALERT_4_G Atom Site Occupancy of H19C       Constrained at       0.65 Check
PLAT300_ALERT_4_G Atom Site Occupancy of H20A       Constrained at       0.65 Check
PLAT300_ALERT_4_G Atom Site Occupancy of H20B       Constrained at       0.65 Check
PLAT300_ALERT_4_G Atom Site Occupancy of H20C       Constrained at       0.65 Check
PLAT300_ALERT_4_G Atom Site Occupancy of H21A       Constrained at       0.65 Check
PLAT300_ALERT_4_G Atom Site Occupancy of H21B       Constrained at       0.65 Check
PLAT300_ALERT_4_G Atom Site Occupancy of H21C       Constrained at       0.65 Check
PLAT300_ALERT_4_G Atom Site Occupancy of H44A       Constrained at        0.7 Check
PLAT300_ALERT_4_G Atom Site Occupancy of H44B       Constrained at        0.7 Check
PLAT300_ALERT_4_G Atom Site Occupancy of H44C       Constrained at        0.7 Check
PLAT300_ALERT_4_G Atom Site Occupancy of H45A       Constrained at        0.7 Check
PLAT300_ALERT_4_G Atom Site Occupancy of H45B       Constrained at        0.7 Check
PLAT300_ALERT_4_G Atom Site Occupancy of H45C       Constrained at        0.7 Check
PLAT300_ALERT_4_G Atom Site Occupancy of H46A       Constrained at        0.7 Check
PLAT300_ALERT_4_G Atom Site Occupancy of H46B       Constrained at        0.7 Check
PLAT300_ALERT_4_G Atom Site Occupancy of H46C       Constrained at        0.7 Check
PLAT300_ALERT_4_G Atom Site Occupancy of H73D       Constrained at        0.6 Check
PLAT300_ALERT_4_G Atom Site Occupancy of H73E       Constrained at        0.6 Check
PLAT300_ALERT_4_G Atom Site Occupancy of H73F       Constrained at        0.6 Check
PLAT300_ALERT_4_G Atom Site Occupancy of H74D       Constrained at        0.6 Check
PLAT300_ALERT_4_G Atom Site Occupancy of H74E       Constrained at        0.6 Check
PLAT300_ALERT_4_G Atom Site Occupancy of H74F       Constrained at        0.6 Check
PLAT300_ALERT_4_G Atom Site Occupancy of H75D       Constrained at        0.6 Check
PLAT300_ALERT_4_G Atom Site Occupancy of H75E       Constrained at        0.6 Check
PLAT300_ALERT_4_G Atom Site Occupancy of H75F       Constrained at        0.6 Check
PLAT300_ALERT_4_G Atom Site Occupancy of H19D       Constrained at       0.35 Check
PLAT300_ALERT_4_G Atom Site Occupancy of H19E       Constrained at       0.35 Check
PLAT300_ALERT_4_G Atom Site Occupancy of H19F       Constrained at       0.35 Check
PLAT300_ALERT_4_G Atom Site Occupancy of H20D       Constrained at       0.35 Check
PLAT300_ALERT_4_G Atom Site Occupancy of H20E       Constrained at       0.35 Check
PLAT300_ALERT_4_G Atom Site Occupancy of H20F       Constrained at       0.35 Check
PLAT300_ALERT_4_G Atom Site Occupancy of H21D       Constrained at       0.35 Check
PLAT300_ALERT_4_G Atom Site Occupancy of H21E       Constrained at       0.35 Check
PLAT300_ALERT_4_G Atom Site Occupancy of H21F       Constrained at       0.35 Check
PLAT300_ALERT_4_G Atom Site Occupancy of H44D       Constrained at        0.3 Check
PLAT300_ALERT_4_G Atom Site Occupancy of H44E       Constrained at        0.3 Check
PLAT300_ALERT_4_G Atom Site Occupancy of H44F       Constrained at        0.3 Check
PLAT300_ALERT_4_G Atom Site Occupancy of H45D       Constrained at        0.3 Check
PLAT300_ALERT_4_G Atom Site Occupancy of H45E       Constrained at        0.3 Check
PLAT300_ALERT_4_G Atom Site Occupancy of H45F       Constrained at        0.3 Check
PLAT300_ALERT_4_G Atom Site Occupancy of H46D       Constrained at        0.3 Check
PLAT300_ALERT_4_G Atom Site Occupancy of H46E       Constrained at        0.3 Check
PLAT300_ALERT_4_G Atom Site Occupancy of H46F       Constrained at        0.3 Check
PLAT300_ALERT_4_G Atom Site Occupancy of H73A       Constrained at        0.4 Check
PLAT300_ALERT_4_G Atom Site Occupancy of H73B       Constrained at        0.4 Check
PLAT300_ALERT_4_G Atom Site Occupancy of H73C       Constrained at        0.4 Check
PLAT300_ALERT_4_G Atom Site Occupancy of H74A       Constrained at        0.4 Check
PLAT300_ALERT_4_G Atom Site Occupancy of H74B       Constrained at        0.4 Check
PLAT300_ALERT_4_G Atom Site Occupancy of H74C       Constrained at        0.4 Check
PLAT300_ALERT_4_G Atom Site Occupancy of H75A       Constrained at        0.4 Check
PLAT300_ALERT_4_G Atom Site Occupancy of H75B       Constrained at        0.4 Check
```

```
PLAT300_ALERT_4_G Atom Site Occupancy of H75C       Constrained at        0.4 Check
PLAT300_ALERT_4_G Atom Site Occupancy of Cl1        Constrained at        0.5 Check
PLAT300_ALERT_4_G Atom Site Occupancy of Cl2        Constrained at        0.5 Check
PLAT300_ALERT_4_G Atom Site Occupancy of C76        Constrained at        0.5 Check
PLAT300_ALERT_4_G Atom Site Occupancy of H76A       Constrained at        0.5 Check
PLAT300_ALERT_4_G Atom Site Occupancy of H76B       Constrained at        0.5 Check
PLAT301_ALERT_3_G Main Residue  Disorder .............(Resd  1  )        14% Note
PLAT302_ALERT_4_G Anion/Solvent/Minor-Residue Disorder (Resd  2  )       100% Note
PLAT304_ALERT_4_G Non-Integer Number of Atoms in ..... (Resd  2  )       2.50 Check
PLAT412_ALERT_2_G Short Intra XH3 .. XHn     H3       ..H20C     .       2.11 Ang.
                                                       x,y,z  =      1_555 Check
PLAT412_ALERT_2_G Short Intra XH3 .. XHn     H28      ..H46F     .       1.89 Ang.
                                                       x,y,z  =      1_555 Check
PLAT860_ALERT_3_G Number of Least-Squares Restraints ............         527 Note
PLAT883_ALERT_1_G No Info/Value for _atom_sites_solution_primary .     Please Do !
PLAT912_ALERT_4_G Missing # of FCF Reflections Above STh/L=  0.600          7 Note
PLAT913_ALERT_3_G Missing # of Very Strong Reflections in FCF ....          1 Note
PLAT933_ALERT_2_G Number of HKL-OMIT Records in Embedded .res File         12 Note
PLAT965_ALERT_2_G The SHELXL WEIGHT Optimisation has not Converged     Please Check
PLAT978_ALERT_2_G Number C-C Bonds with Positive Residual Density.          1 Info
```

```
  0 ALERT level A = Most likely a serious problem - resolve or explain
  0 ALERT level B = A potentially serious problem, consider carefully
 17 ALERT level C = Check. Ensure it is not caused by an omission or oversight
105 ALERT level G = General information/check it is not something unexpected

  3 ALERT type 1 CIF construction/syntax error, inconsistent or missing data
 19 ALERT type 2 Indicator that the structure model may be wrong or deficient
  6 ALERT type 3 Indicator that the structure quality may be low
 94 ALERT type 4 Improvement, methodology, query or suggestion
  0 ALERT type 5 Informative message, check
```

Publication of your CIF

A full structural check has been run on your CIF. This includes checks on:

- CIF syntax and construction
- Cell and geometry details
- Space-group symmetry
- Anisotropic displacement parameters

Structure-factor checking is currently being tested on articles submitted to *Acta Crystallographica Section C* and *Acta Crystallographica Section E*. These tests may be carried out with a local version of PLATON or the trial service here.

These full checks give an indication of potential problems with your CIF. Please note that if you intend to submit your CIF for publication in Acta Crystallographica Section C or E or IUCrData, you must make sure that full publication checks are run on the final version of the CIF prior to submission.

If you intend to submit to another section of Acta Crystallographica, Journal of Applied Crystallography or Journal of Synchrotron Radiation, you should make sure that at least basic structural checks are run on the final version of your CIF prior to submission.

To submit your CIF for publication in an IUCr journal click here.

PLATON version of 19/02/2022; check.def file version of 19/01/2022

Datablock 1_a - ellipsoid plot

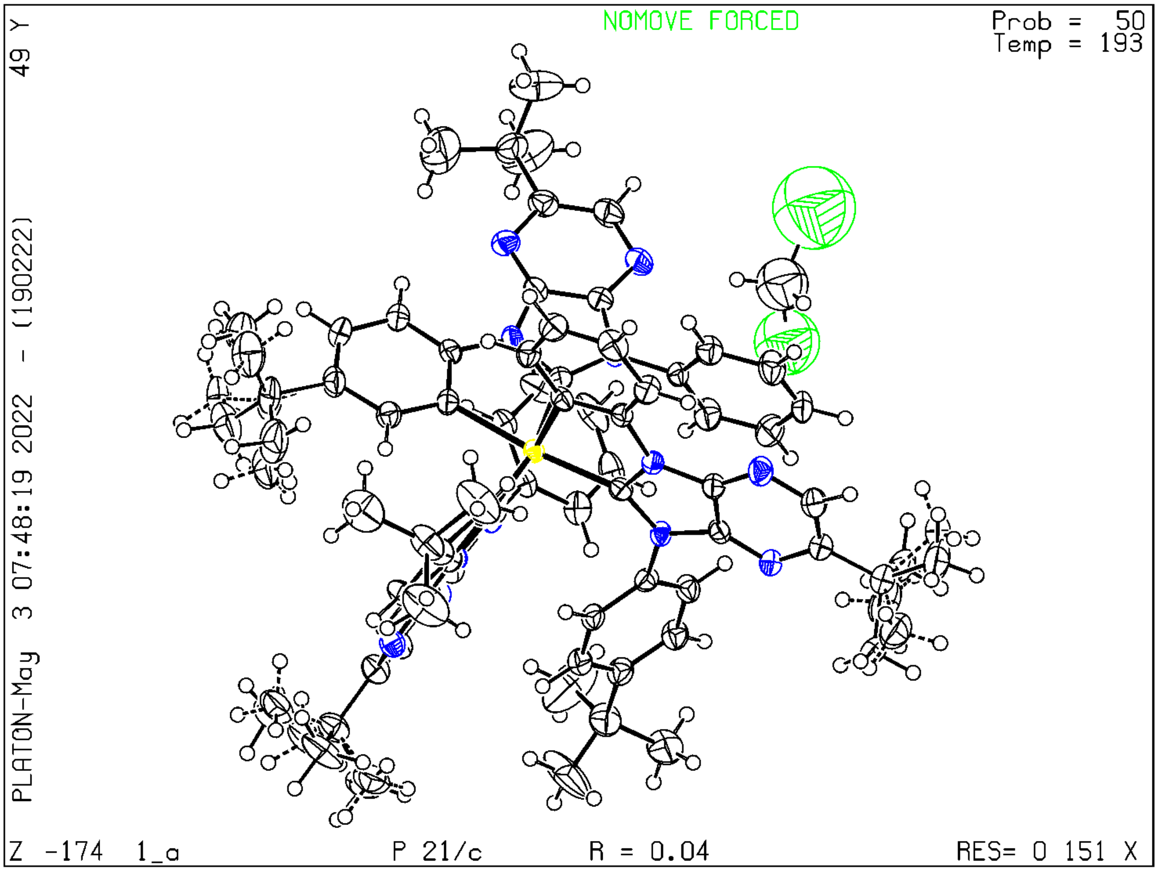

Supplement: Supplementary file 5 — Supplementary Data 2 [file 41467_2023_42090_MOESM5_ESM.pdf]
