# Supplementary material for: Electroluminescence and hyperphosphorescence from stable blue Ir(III) carbene complexes with suppressed efficiency roll-off
Source: Nat Commun. 2023 Oct 12;14:6419. doi: 10.1038/s41467-023-42090-z (PMC10570383; doi:10.1038/s41467-023-42090-z)

checkCIF/PLATON report

Structure factors have been supplied for datablock(s) 1_a

THIS REPORT IS FOR GUIDANCE ONLY. IF USED AS PART OF A REVIEW PROCEDURE FOR PUBLICATION, IT SHOULD NOT REPLACE THE EXPERTISE OF AN EXPERIENCED CRYSTALLOGRAPHIC REFEREE.

No syntax errors found. CIF dictionary Interpreting this report

Datablock: 1_a

Bond precision: C-C = 0.0048 A Wavelength=0.71073

Cell:	a=13.1528(4)	b=14.6232(5)	c=19.0628(6)
	alpha=84.952(1)	beta=88.844(1)	gamma=89.291(1)
Temperature:	193 K		

	Calculated	Reported
Volume	3651.3(2)	3651.3(2)
Space group	P -1	P -1
Hall group	-P 1	-P 1
Moiety formula	C75 H81 Ir N12, 2(C H2 Cl2)	?
Sum formula	C77 H85 Cl4 Ir N12	C77 H85 Cl4 Ir N12
Mr	1512.60	1512.56
Dx,g cm-3	1.376	1.376
Z	2	2
Mu (mm-1)	2.026	2.026
F000	1552.0	1552.0
F000'	1550.70	
h,k,lmax	16,18,23	16,18,23
Nref	14957	14929
Tmin,Tmax	0.606,0.941	0.642,0.745
Tmin'	0.414	

Correction method= # Reported T Limits: Tmin=0.642 Tmax=0.745
AbsCorr = MULTI-SCAN

Data completeness= 0.998 Theta(max)= 26.382

R(reflections)= 0.0297(13595) wR2(reflections)= 0.0738(14929)

S = 1.030 Npar= 892

The following ALERTS were generated. Each ALERT has the format
test-name_ALERT_alert-type_alert-level.
Click on the hyperlinks for more details of the test.

Alert level C

```
PLAT048_ALERT_1_C MoietyFormula Not Given (or Incomplete) ........     Please Check
PLAT213_ALERT_2_C Atom C48             has ADP max/min Ratio .....        3.1 prolat
PLAT220_ALERT_2_C NonSolvent   Resd 1  C   Ueq(max)/Ueq(min) Range        5.3 Ratio
PLAT222_ALERT_3_C NonSolvent Resd 1  H   Uiso(max)/Uiso(min) Range        5.7 Ratio
PLAT242_ALERT_2_C Low    'MainMol' Ueq as Compared to Neighbors of        C18 Check
PLAT242_ALERT_2_C Low    'MainMol' Ueq as Compared to Neighbors of        C43 Check
PLAT242_ALERT_2_C Low    'MainMol' Ueq as Compared to Neighbors of        C47 Check
PLAT244_ALERT_4_C Low    'Solvent' Ueq as Compared to Neighbors of        C76 Check
PLAT260_ALERT_2_C Large Average Ueq of Residue Including      Cl3A      0.115 Check
PLAT601_ALERT_2_C Unit Cell Contains Solvent Accessible VOIDS of .         32 Ang**3
PLAT910_ALERT_3_C Missing # of FCF Reflection(s) Below Theta(Min).         10 Note
PLAT911_ALERT_3_C Missing FCF Refl Between Thmin & STh/L=    0.600          2 Report
PLAT934_ALERT_3_C Number of (Iobs-Icalc)/Sigma(W) > 10 Outliers ..          1 Check
PLAT977_ALERT_2_C Check Negative Difference Density on H77C      .      -0.32 eA-3
```

Alert level G

```
PLAT002_ALERT_2_G Number of Distance or Angle Restraints on AtSite          6 Note
PLAT003_ALERT_2_G Number of Uiso or Uij Restrained non-H Atoms ...          6 Report
PLAT154_ALERT_1_G The s.u.'s on the Cell Angles are Equal ..(Note)      0.001 Degree
PLAT172_ALERT_4_G The CIF-Embedded .res File Contains DFIX Records          2 Report
PLAT177_ALERT_4_G The CIF-Embedded .res File Contains DELU Records          1 Report
PLAT178_ALERT_4_G The CIF-Embedded .res File Contains SIMU Records          1 Report
PLAT186_ALERT_4_G The CIF-Embedded .res File Contains ISOR Records          1 Report
PLAT232_ALERT_2_G Hirshfeld Test Diff (M-X)  Ir1      --C1       .        5.4 s.u.
PLAT232_ALERT_2_G Hirshfeld Test Diff (M-X)  Ir1      --C26      .        5.4 s.u.
PLAT300_ALERT_4_G Atom Site Occupancy of Cl3        Constrained at        0.7 Check
PLAT300_ALERT_4_G Atom Site Occupancy of Cl4        Constrained at        0.7 Check
PLAT300_ALERT_4_G Atom Site Occupancy of C77        Constrained at        0.7 Check
PLAT300_ALERT_4_G Atom Site Occupancy of H77A       Constrained at        0.7 Check
PLAT300_ALERT_4_G Atom Site Occupancy of H77B       Constrained at        0.7 Check
PLAT300_ALERT_4_G Atom Site Occupancy of Cl3A       Constrained at        0.3 Check
PLAT300_ALERT_4_G Atom Site Occupancy of Cl4A       Constrained at        0.3 Check
PLAT300_ALERT_4_G Atom Site Occupancy of C77A       Constrained at        0.3 Check
PLAT300_ALERT_4_G Atom Site Occupancy of H77C       Constrained at        0.3 Check
PLAT300_ALERT_4_G Atom Site Occupancy of H77D       Constrained at        0.3 Check
PLAT302_ALERT_4_G Anion/Solvent/Minor-Residue Disorder (Resd  3  )       100% Note
PLAT302_ALERT_4_G Anion/Solvent/Minor-Residue Disorder (Resd  4  )       100% Note
PLAT304_ALERT_4_G Non-Integer Number of Atoms in ..... (Resd  3  )       3.50 Check
PLAT304_ALERT_4_G Non-Integer Number of Atoms in ..... (Resd  4  )       1.50 Check
PLAT860_ALERT_3_G Number of Least-Squares Restraints .............        106 Note
PLAT883_ALERT_1_G No Info/Value for _atom_sites_solution_primary .     Please Do !
PLAT912_ALERT_4_G Missing # of FCF Reflections Above STh/L=  0.600         16 Note
PLAT941_ALERT_3_G Average HKL Measurement Multiplicity ..........         3.8 Low
PLAT965_ALERT_2_G The SHELXL WEIGHT Optimisation has not Converged     Please Check
PLAT978_ALERT_2_G Number C-C Bonds with Positive Residual Density.          1 Info
```

0 **ALERT level A** = Most likely a serious problem - resolve or explain
0 **ALERT level B** = A potentially serious problem, consider carefully

```
14 ALERT level C = Check. Ensure it is not caused by an omission or oversight
29 ALERT level G = General information/check it is not something unexpected

 3 ALERT type 1 CIF construction/syntax error, inconsistent or missing data
14 ALERT type 2 Indicator that the structure model may be wrong or deficient
 6 ALERT type 3 Indicator that the structure quality may be low
20 ALERT type 4 Improvement, methodology, query or suggestion
 0 ALERT type 5 Informative message, check
```

Publication of your CIF

A full structural check has been run on your CIF. This includes checks on:

- CIF syntax and construction
- Cell and geometry details
- Space-group symmetry
- Anisotropic displacement parameters

Structure-factor checking is currently being tested on articles submitted to *Acta Crystallographica Section C* and *Acta Crystallographica Section E*. These tests may be carried out with a local version of PLATON or the trial service here.

These full checks give an indication of potential problems with your CIF. Please note that if you intend to submit your CIF for publication in Acta Crystallographica Section C or E or IUCrData, you must make sure that full publication checks are run on the final version of the CIF prior to submission.

If you intend to submit to another section of Acta Crystallographica, Journal of Applied Crystallography or Journal of Synchrotron Radiation, you should make sure that at least basic structural checks are run on the final version of your CIF prior to submission.

To submit your CIF for publication in an IUCr journal click here.

PLATON version of 19/02/2022; check.def file version of 19/01/2022

Datablock 1_a - ellipsoid plot

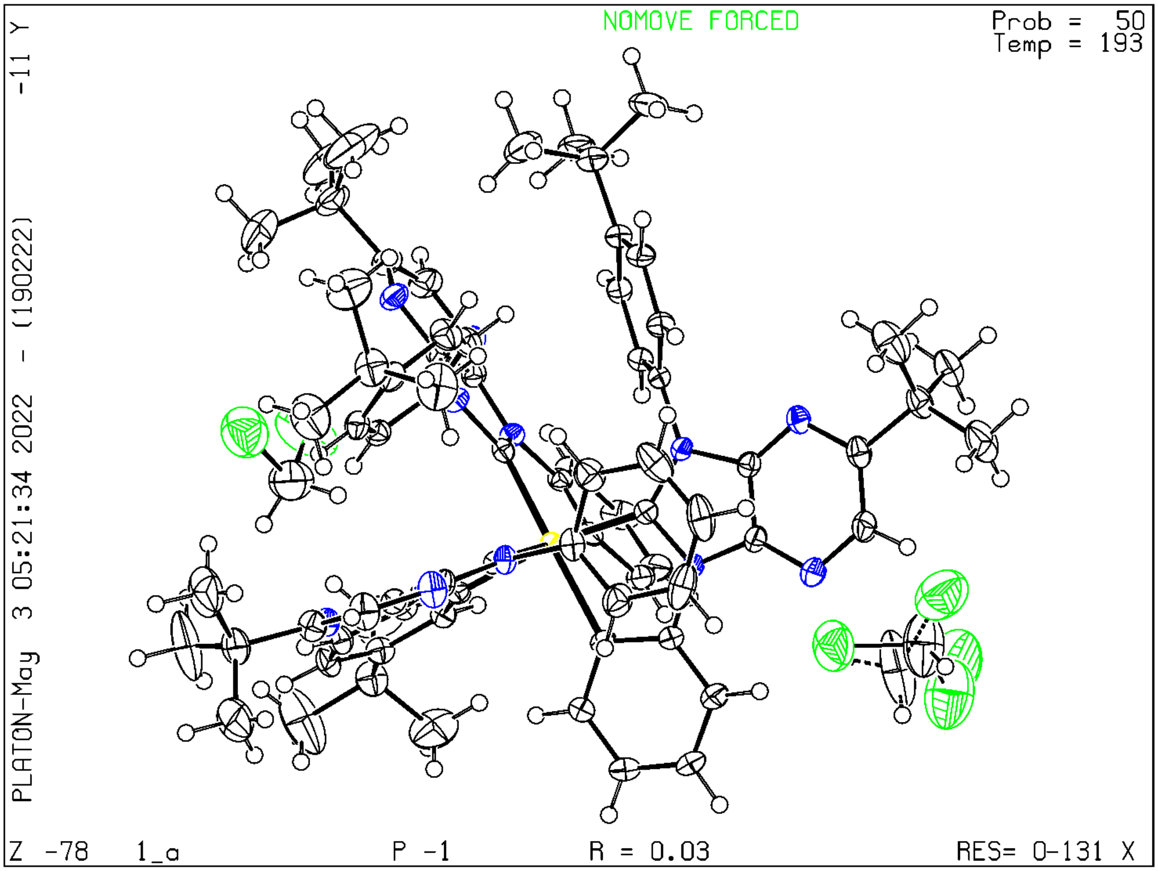

Supplement: Supplementary file 6 — Supplementary Data 3 [file 41467_2023_42090_MOESM6_ESM.pdf]
