# Supplementary material for: Electroluminescence and hyperphosphorescence from stable blue Ir(III) carbene complexes with suppressed efficiency roll-off
Source: Nat Commun. 2023 Oct 12;14:6419. doi: 10.1038/s41467-023-42090-z (PMC10570383; doi:10.1038/s41467-023-42090-z)

checkCIF/PLATON report

Structure factors have been supplied for datablock(s) 1_a

THIS REPORT IS FOR GUIDANCE ONLY. IF USED AS PART OF A REVIEW PROCEDURE FOR PUBLICATION, IT SHOULD NOT REPLACE THE EXPERTISE OF AN EXPERIENCED CRYSTALLOGRAPHIC REFEREE.

No syntax errors found. CIF dictionary Interpreting this report

Datablock: 1_a

Bond precision: C-C = 0.0124 A Wavelength=1.54178

Cell:	a=31.3192(6)	b=31.3192(6)	c=31.3192(6)
	alpha=90	beta=90	gamma=90
Temperature:	193 K		

	Calculated	Reported
Volume	30720.8(18)	30720.8(18)
Space group	I -4 3 d	I -4 3 d
Hall group	I -4bd 2c 3	I -4bd 2c 3
Moiety formula	2(C75 H81 Ir N12), C H Cl3, 3(C0.33 H0.33 Cl)	?
Sum formula	C152 H164 Cl6 Ir2 N24	C76 H82 Cl3 Ir N12
Mr	2924.24	1462.08
Dx,g cm-3	1.265	1.264
Z	8	16
Mu (mm-1)	4.692	4.692
F000	12000.1	12000.0
F000'	11967.37	
h,k,lmax	39,39,39	39,39,38
Nref	5277[2789]	5253
Tmin,Tmax	0.467,0.829	0.531,0.754
Tmin'	0.121	

Correction method= # Reported T Limits: Tmin=0.531 Tmax=0.754
AbsCorr = MULTI-SCAN

Data completeness= 1.88/1.00 Theta(max)= 74.861

R(reflections)= 0.0401(5014) wR2(reflections)= 0.1105(5253)

S = 1.048 Npar= 359

The following ALERTS were generated. Each ALERT has the format

test-name_ALERT_alert-type_alert-level.

Click on the hyperlinks for more details of the test.

Alert level C

```
PLAT090_ALERT_3_C Poor Data / Parameter Ratio (Zmax > 18) ........       7.74 Note
PLAT242_ALERT_2_C Low    'MainMol' Ueq as Compared to Neighbors of        C22 Check
PLAT260_ALERT_2_C Large Average Ueq of Residue Including       Cl2      0.141 Check
PLAT260_ALERT_2_C Large Average Ueq of Residue Including       Cl1      0.132 Check
PLAT342_ALERT_3_C Low Bond Precision on  C-C Bonds ...............    0.01244 Ang.
PLAT601_ALERT_2_C Unit Cell Contains Solvent Accessible VOIDS of .         77 Ang**3
PLAT911_ALERT_3_C Missing FCF Refl Between Thmin & STh/L=    0.600          3 Report
```

Alert level G

```
PLAT002_ALERT_2_G Number of Distance or Angle Restraints on AtSite         13 Note
PLAT003_ALERT_2_G Number of Uiso or Uij Restrained non-H Atoms ...         14 Report
PLAT045_ALERT_1_G Calculated and Reported Z Differ by a Factor ...      0.500 Check
PLAT083_ALERT_2_G SHELXL Second Parameter in WGHT  Unusually Large      15.51 Why ?
PLAT172_ALERT_4_G The CIF-Embedded .res File Contains DFIX Records          4 Report
PLAT173_ALERT_4_G The CIF-Embedded .res File Contains DANG Records          3 Report
PLAT177_ALERT_4_G The CIF-Embedded .res File Contains DELU Records          2 Report
PLAT178_ALERT_4_G The CIF-Embedded .res File Contains SIMU Records          2 Report
PLAT186_ALERT_4_G The CIF-Embedded .res File Contains ISOR Records          2 Report
PLAT300_ALERT_4_G Atom Site Occupancy of C12         Constrained at       0.5 Check
PLAT300_ALERT_4_G Atom Site Occupancy of C12A        Constrained at       0.5 Check
PLAT300_ALERT_4_G Atom Site Occupancy of C13         Constrained at       0.5 Check
PLAT300_ALERT_4_G Atom Site Occupancy of C13A        Constrained at       0.5 Check
PLAT300_ALERT_4_G Atom Site Occupancy of C14         Constrained at       0.5 Check
PLAT300_ALERT_4_G Atom Site Occupancy of C14A        Constrained at       0.5 Check
PLAT300_ALERT_4_G Atom Site Occupancy of C15         Constrained at       0.5 Check
PLAT300_ALERT_4_G Atom Site Occupancy of C15A        Constrained at       0.5 Check
PLAT300_ALERT_4_G Atom Site Occupancy of H13A        Constrained at       0.5 Check
PLAT300_ALERT_4_G Atom Site Occupancy of H13B        Constrained at       0.5 Check
PLAT300_ALERT_4_G Atom Site Occupancy of H13C        Constrained at       0.5 Check
PLAT300_ALERT_4_G Atom Site Occupancy of H13D        Constrained at       0.5 Check
PLAT300_ALERT_4_G Atom Site Occupancy of H13E        Constrained at       0.5 Check
PLAT300_ALERT_4_G Atom Site Occupancy of H13F        Constrained at       0.5 Check
PLAT300_ALERT_4_G Atom Site Occupancy of H14A        Constrained at       0.5 Check
PLAT300_ALERT_4_G Atom Site Occupancy of H14B        Constrained at       0.5 Check
PLAT300_ALERT_4_G Atom Site Occupancy of H14C        Constrained at       0.5 Check
PLAT300_ALERT_4_G Atom Site Occupancy of H14D        Constrained at       0.5 Check
PLAT300_ALERT_4_G Atom Site Occupancy of H14E        Constrained at       0.5 Check
PLAT300_ALERT_4_G Atom Site Occupancy of H14F        Constrained at       0.5 Check
PLAT300_ALERT_4_G Atom Site Occupancy of H15A        Constrained at       0.5 Check
PLAT300_ALERT_4_G Atom Site Occupancy of H15B        Constrained at       0.5 Check
PLAT300_ALERT_4_G Atom Site Occupancy of H15C        Constrained at       0.5 Check
PLAT300_ALERT_4_G Atom Site Occupancy of H15D        Constrained at       0.5 Check
PLAT300_ALERT_4_G Atom Site Occupancy of H15E        Constrained at       0.5 Check
PLAT300_ALERT_4_G Atom Site Occupancy of H15F        Constrained at       0.5 Check
PLAT300_ALERT_4_G Atom Site Occupancy of Cl2         Constrained at    0.1667 Check
PLAT300_ALERT_4_G Atom Site Occupancy of Cl3         Constrained at    0.1667 Check
PLAT300_ALERT_4_G Atom Site Occupancy of Cl4         Constrained at    0.1667 Check
PLAT300_ALERT_4_G Atom Site Occupancy of C27         Constrained at    0.1667 Check
PLAT300_ALERT_4_G Atom Site Occupancy of H27         Constrained at    0.1667 Check
```

```
PLAT300_ALERT_4_G Atom Site Occupancy of Cl1       Constrained at        0.5 Check
PLAT300_ALERT_4_G Atom Site Occupancy of C26       Constrained at        0.5 Check
PLAT300_ALERT_4_G Atom Site Occupancy of H26       Constrained at     0.5001 Check
PLAT301_ALERT_3_G Main Residue  Disorder .............(Resd  1  )        14% Note
PLAT302_ALERT_4_G Anion/Solvent/Minor-Residue Disorder (Resd  2  )       100% Note
PLAT302_ALERT_4_G Anion/Solvent/Minor-Residue Disorder (Resd  3  )       100% Note
PLAT304_ALERT_4_G Non-Integer Number of Atoms in ..... (Resd  2  )       0.83 Check
PLAT304_ALERT_4_G Non-Integer Number of Atoms in ..... (Resd  3  )       0.83 Check
PLAT412_ALERT_2_G Short Intra XH3 .. XHn     H8       ..H13E     .       2.13 Ang.
                                                    x,y,z  =      1_555 Check
PLAT789_ALERT_4_G Atoms with Negative _atom_site_disorder_group  #          5 Check
PLAT860_ALERT_3_G Number of Least-Squares Restraints ............         249 Note
PLAT883_ALERT_1_G No Info/Value for _atom_sites_solution_primary .     Please Do !
PLAT912_ALERT_4_G Missing # of FCF Reflections Above STh/L=  0.600          5 Note
PLAT933_ALERT_2_G Number of HKL-OMIT Records in Embedded .res File          4 Note
PLAT955_ALERT_1_G Reported (CIF) and Actual (FCF) Lmax Differ by .          1 Units
PLAT965_ALERT_2_G The SHELXL WEIGHT Optimisation has not Converged     Please Check
PLAT978_ALERT_2_G Number C-C Bonds with Positive Residual Density.          0 Info
```

---

0 **ALERT level A** = Most likely a serious problem - resolve or explain
0 **ALERT level B** = A potentially serious problem, consider carefully
7 **ALERT level C** = Check. Ensure it is not caused by an omission or oversight
57 **ALERT level G** = General information/check it is not something unexpected

3 ALERT type 1 CIF construction/syntax error, inconsistent or missing data
11 ALERT type 2 Indicator that the structure model may be wrong or deficient
5 ALERT type 3 Indicator that the structure quality may be low
45 ALERT type 4 Improvement, methodology, query or suggestion
0 ALERT type 5 Informative message, check

Validation response form

Please find below a validation response form (VRF) that can be filled in and pasted into your CIF.

```
# start Validation Reply Form
_vrf_PLAT090_1_a
;
PROBLEM: Poor Data / Parameter Ratio (Zmax > 18) ........       7.74 Note
RESPONSE: ...
;
_vrf_PLAT242_1_a
;
PROBLEM: Low    'MainMol' Ueq as Compared to Neighbors of        C22 Check
RESPONSE: ...
;
_vrf_PLAT260_1_a
;
PROBLEM: Large Average Ueq of Residue Including       Cl2      0.141 Check
RESPONSE: ...
;
_vrf_PLAT342_1_a
;
PROBLEM: Low Bond Precision on  C-C Bonds ..............    0.01244 Ang.
RESPONSE: ...
;
```

```
_vrf_PLAT601_1_a
;
PROBLEM: Unit Cell Contains Solvent Accessible VOIDS of .         77 Ang**3
RESPONSE: ...
;
_vrf_PLAT911_1_a
;
PROBLEM: Missing FCF Refl Between Thmin & STh/L=    0.600          3 Report
RESPONSE: ...
;
# end Validation Reply Form
```

It is advisable to attempt to resolve as many as possible of the alerts in all categories. Often the minor alerts point to easily fixed oversights, errors and omissions in your CIF or refinement strategy, so attention to these fine details can be worthwhile. In order to resolve some of the more serious problems it may be necessary to carry out additional measurements or structure refinements. However, the purpose of your study may justify the reported deviations and the more serious of these should normally be commented upon in the discussion or experimental section of a paper or in the "special_details" fields of the CIF. checkCIF was carefully designed to identify outliers and unusual parameters, but every test has its limitations and alerts that are not important in a particular case may appear. Conversely, the absence of alerts does not guarantee there are no aspects of the results needing attention. It is up to the individual to critically assess their own results and, if necessary, seek expert advice.

Publication of your CIF in IUCr journals

A basic structural check has been run on your CIF. These basic checks will be run on all CIFs submitted for publication in IUCr journals (*Acta Crystallographica*, *Journal of Applied Crystallography*, *Journal of Synchrotron Radiation*); however, if you intend to submit to *Acta Crystallographica Section C* or *E* or *IUCrData*, you should make sure that full publication checks are run on the final version of your CIF prior to submission.

Publication of your CIF in other journals

Please refer to the *Notes for Authors* of the relevant journal for any special instructions relating to CIF submission.

PLATON version of 18/05/2022; check.def file version of 17/05/2022

Datablock 1_a - ellipsoid plot

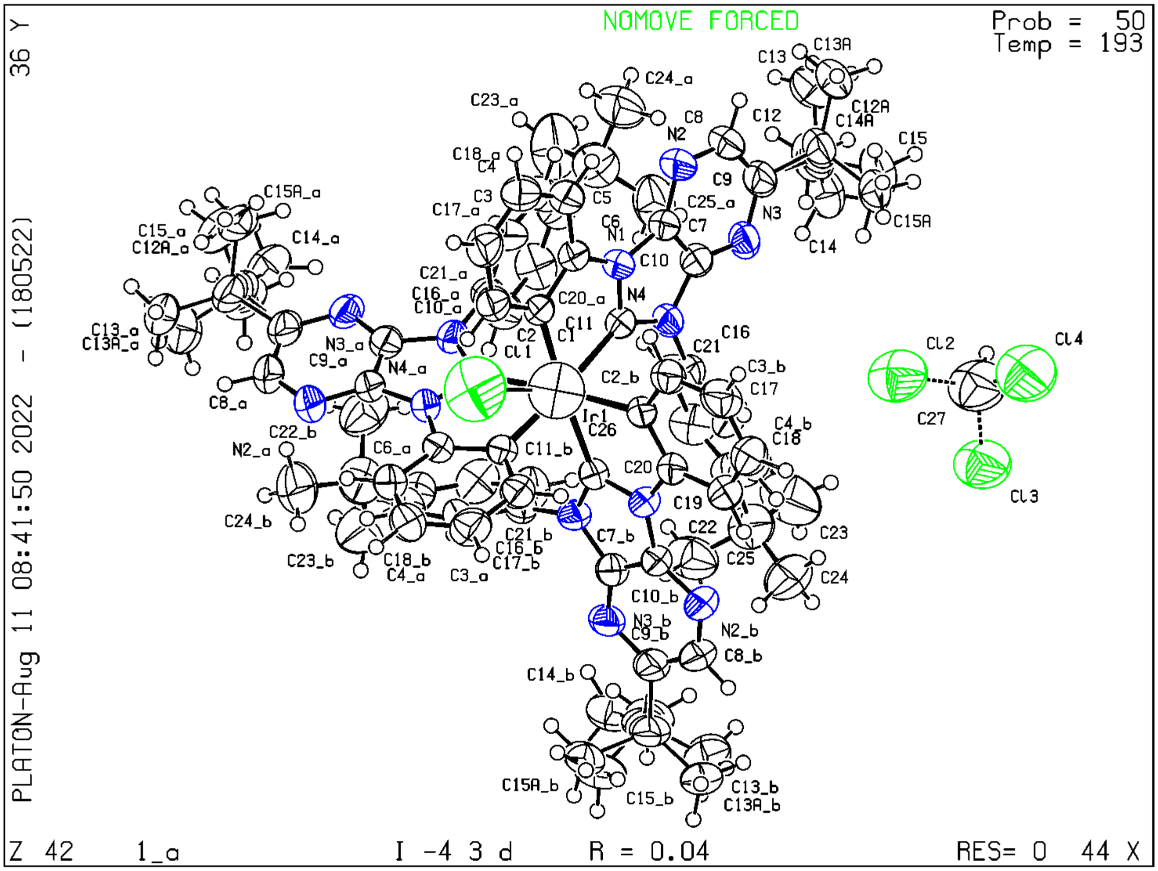

Supplement: Supplementary file 7 — Supplementary Data 4 [file 41467_2023_42090_MOESM7_ESM.pdf]
